# Constitutive expression of a pea apyrase, *psNTP9*, increases seed yield in field-grown soybean

**DOI:** 10.1038/s41598-022-14821-7

**Published:** 2022-06-27

**Authors:** Tanya Sabharwal, Zhongjin Lu, Robert D. Slocum, Seongjoon Kang, Huan Wang, Han-Wei Jiang, Roopadarshini Veerappa, Dwight Romanovicz, Ji Chul Nam, Simon Birk, Greg Clark, Stanley J. Roux

**Affiliations:** 1 Department of Molecular Biosciences, The University of Texas at Austin, Austin, TX 78712 USA; 2 Texas Crop Science, Inc., Austin, TX USA; 3 Program in Biological Sciences, Goucher College, Towson, MD 21204 USA

**Keywords:** Plant biotechnology, Field trials, Biotechnology, Plant sciences

## Abstract

To address the demand for food by a rapidly growing human population, agricultural scientists have carried out both plant breeding and genetic engineering research. Previously, we reported that the constitutive expression of a pea apyrase (Nucleoside triphosphate, diphosphohydrolase) gene, *psNTP9*, under the control of the CaMV35S promoter, resulted in soybean plants with an expanded root system architecture, enhanced drought resistance and increased seed yield when they are grown in greenhouses under controlled conditions. Here, we report that *psNTP9*-expressing soybean lines also show significantly enhanced seed yields when grown in multiple different field conditions at multiple field sites, including when the gene is introgressed into elite germplasm. The transgenic lines have higher leaf chlorophyll and soluble protein contents and decreased stomatal density and cuticle permeability, traits that increase water use efficiency and likely contribute to the increased seed yields of field-grown plants. These altered properties are explained, in part, by genome-wide gene expression changes induced by the transgene.

## Introduction

The importance of increasing agriculture yields in order to meet the needs of a growing world population has long been recognized^1^. Efforts to improve crop yields using genetic approaches provide a clear advantage over traditional breeding and are beginning to provide examples of increased yield in a variety of crops^2^. One recent study found that overexpression of transcription factor zmm28 led to an increase in the yield of corn in field trials^3^ and another corn study showed that overexpression of a protein kinase KERNEL NUMBER PER ROW6 (KNR6) increased ear length and kernel row number^4^. In soybeans, the constitutive expression of the maize *SOC1* gene increased the seed yield in this crop^5^. As an example of the value of the transgenic approach in other crops, expressing a human RNA demethylase in rice and potato increased the yield of these crops^6^. There are also multiple projects aimed at using genetic engineering to improve photosynthesis in plants^7,8^.

Genes that control plant growth would be good candidates to test in transgenic approaches to increase crop productivity. In this regard, numerous studies have revealed that apyrase (APY) enzymes, which, like their NTPDase orthologues in animals, help control the concentration of extracellular ATP [eATP], play key roles in the control of plant growth and development^9–11^. In Arabidopsis, the overexpression of *AtAPY1* promotes the transport of the growth hormone, auxin, and seedling growth, while the suppression of this enzyme inhibits auxin transport and suppresses growth^12,13^. Higher levels of *APY* expression are positively linked to increased growth in diverse tissues of Arabidopsis, including seedling shoots and roots^14^, pollen tubes^15^, hypocotyls^16^, primary roots^16,17^ and root hairs^18,19^. Apyrase expression also has a positive impact on growth in other plants^11^, such as in potatoes^20^ and cotton fibers^21^. Early studies indicated there was likely a link between apyrase control of growth and its control of eATP, because changing the concentration of applied ATP altered the transport of auxin in seedlings^22^ and regulated plant-cell growth in a biphasic fashion: low levels promoted the growth of pollen, root hairs and cotton fibers while high levels inhibited their growth^18,21,23^.

An apyrase from peas, psNTP9 (also named psAPY1), was one of the first plant apyrases to be well characterized. The early work focused on the nuclear function of this apyrase^24,25^, while later studies indicated that the pea apyrase also functions as an ectoapyrase in the extracellular matrix (ECM), where it could mobilize phosphate from eATP^14^. By promoting phosphate uptake, *psNTP9* complemented a yeast *pho84* mutant defective in phosphate transport, while ectopic expression of this apyrase in Arabidopsis resulted in improved leaf growth in seedlings grown on defined media on plates^14^. Notably, psNTP9 can bind calmodulin which would make it more responsive to eATP-induced changes in [Ca^2+^]_cyt_^24^.

The early results showing a link between eATP and apyrase levels with auxin transport and growth in Arabidopsis led to tests of the impact of genetically enhanced apyrase expression in crop plants. In greenhouse-grown Williams 82 soybean plants, the ectopic expression of *psNTP9* resulted in an improved root system architecture and increased yields under both normal and drought conditions^19^. Enhancing root system architecture often contributes to increased crop yield^26–28^. The transgenic soybean lines used in the greenhouse study are more specifically identified here as 14A, 16C, and 17B, respectively. This present study is an extension of those greenhouse findings and examines the yield performance of soybean plants overexpressing the pea apyrase in field trials. We also address the question of whether the positive effects on soybean yields occurs when the pea apyrase is introgressed into different elite soybean lines and grown in field trials.

## Results

### Genomic insertion of *psNTP9* in three soybean lines

**Figure 1:**
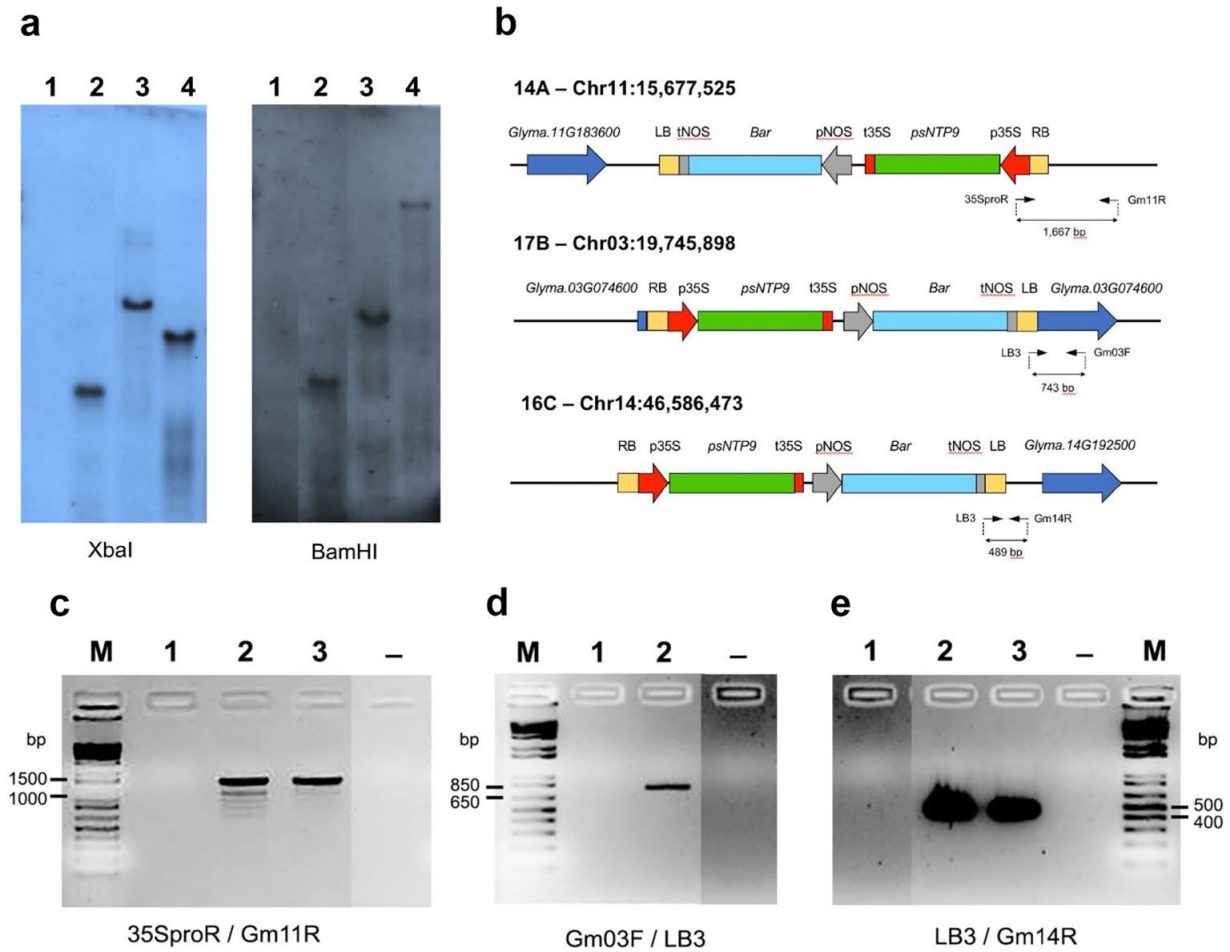
Identification of T-DNA copy number and insertion sites of transgenic soybean lines. (**a**) Southern blot analysis to determine the T-DNA copy number of soybean transgenic lines. Genomic DNA was digested with XbaI or BamHI, and a probe specific to the *Bar* gene was used to detect T-DNA insertions. (**b**) Diagrams of the sites of T-DNA insertions, including their adjacent genes, identified by TAIL-PCR and inverse PCR. Each of the inserted loci is shown as a chromosome number with the location in the chromosome. The locations of primers used for PCR validation of T-DNA insertion sites and the sizes of the resulting products are shown. Some parts of the diagram are not drawn to scale. (**c**–**e**) PCR validation of insertion sites for transgenic soybean lines. M: 1 Kb Plus DNA Ladder (Thermo Fisher Scientific), 1: negative control of non-transgenic soybean; 2, 3: transgenic plants of 14A (**c**), 17B (**d**), and 16C (**e**); –: distilled water control.

Southern blot analysis was performed with a probe specific to Bar gene in the T-DNA construct to determine the T-DNA copy number in the three independent transgenic lines of *Glycine max* analyzed, 14A, 16C, and 17B. Southern blot hybridizations with the genomic DNA cut by XbaI or BamHI indicated that a single copy of T-DNA was integrated into the chromosomes in each transgenic line (Fig. 1a). Afterward, two PCR-based methods of TAIL-PCR and inverse PCR were conducted to identify the insertion loci of the T-DNA in each line. The sequences of the resultant TAIL-PCR and inverse PCR amplicons indicated the integration sites of 14A, 17B, and 16C. Blastn queries of the sequence data against soybean reference genome revealed the T-DNA was integrated at a position around Gm11: 15,677,525 in 14A line, Gm03: 19,745,898 in 17B line, and Gm14: 46,586,473 in 16C line (Fig. 1b). T-DNA insertion orientation was reverse in 17B line and forward in 14A and 16C lines. The T-DNA constructs in 14A and 16C were inserted in intergenic regions of the soybean genome, therefore, no endogenous genes were directly disrupted by the integrations of the T-DNA in those lines. In the 17B line, the T-DNA was located at + 13 bp position, within the 5′-UTR of the *Glyma.03G074600* gene model but was located 131 bp upstream of the LOC100499762 transcription start site. The latter gene model has stronger support from RNA-seq reads coverage.

The T-DNA Insertion sites were confirmed by PCR amplification. Primers were designed to amplify the fragments that span the T-DNA region and the upstream or downstream flanking region. The expected amplicon sizes of the primer set of 35SproR/Gm11R for 14A, Gm03F/LB3 for 17B, and LB3/GM14R for 16C were 1,667 bp, 743 bp, and 489 bp respectively based on the integration sites in each transgenic line (Fig. 1b). The PCR size product from three transgenic soybean lines matched the expected product length (Fig. 1c–e). These results further confirmed the T-DNA insertion loci in the genome of each transgenic line.

We identified the genes neighboring the single insertion site for all three transgenic lines. For 17B, the two neighboring genes were *Glyma.3G074700*, encoding a predicted membrane protein involved in ER to Golgi vesicle-mediated transport, and *Glyma.3G074600*, which encodes an F-box/RNI-like superfamily protein subunit of the SCF complex that functions in ubiquitin-mediated protein degradation^29^. Neither gene was differentially expressed (DE) in the transgenic line. For 16C, the closest neighboring gene to the insertion site was *Glyma.14G192500*, encoding Cytochrome P450 86A1, which functions in fatty acid metabolism and suberin synthesis in the root^30^. This gene also was not DE. For 14A, there were two neighboring genes. *Glyma.11G183600*, whose 3′-end is 2716 bp upstream of the insert, encodes an F-box/LRR-repeat protein.

*Glyma.11G183700* encodes the bZIP TF GmTGA14, whose closest Arabidopsis ortholog is *TGA9*. Although the 5′-end of *Glyma.11G183700* is located 9583 bp downstream of the T-DNA insertion, its expression was strongly upregulated (13.5-fold) in 14A leaves. In fact, it was the most highly DE gene in leaf tissues. qRT-PCR analyses showed a 21.7-fold increase in expression of this gene, confirming transcriptome data (Supplementary Table S2). In contrast, *Glyma.11G183700* expression in 17B increased 1.9-fold (Supplementary Table S2).

### Seed yield performance of transgenic events expressing *psNTP9* in field trials

**Figure 2:**
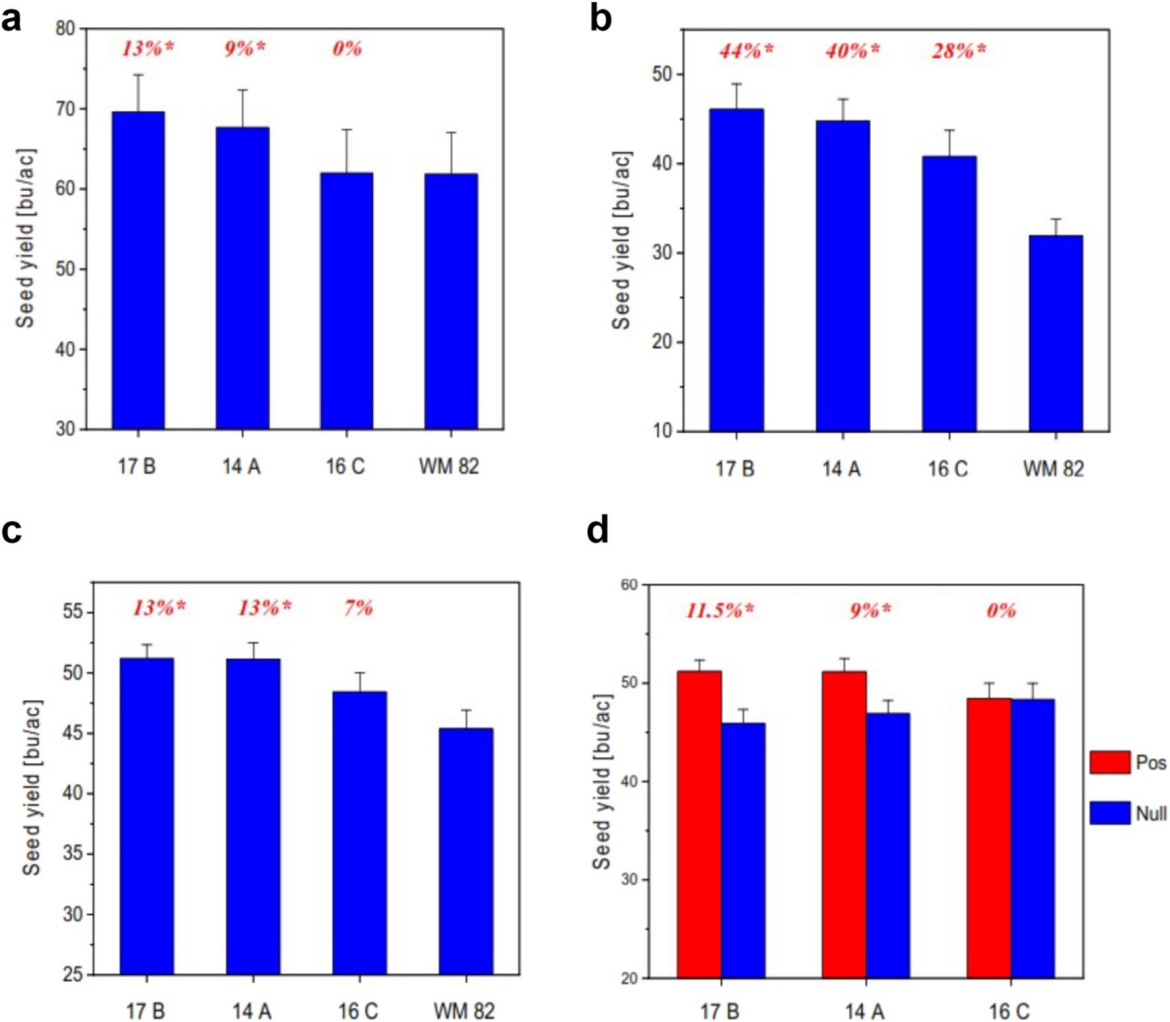
Seed yield of *psNTP9* events and parental line Williams 82 (WM 82) trialed in 2016, 2017, and 2018. (**a**) Field trials were conducted at three different locations in the US—El Paso, Illinois; Henderson, Kentucky; and Atlanta, Indiana. Seed yield performance and analysis of *psNTP9* events at each of the individual sites can be found in supplemental data. Combined site analysis over the three trials determined 5.25 bushels per acre as the value of 5% least squared difference (LSD_0.05_) for mean separations. Asterisk symbol (*) next to yield delta (%) inside the figure indicates significant yield difference from parental line WM 82 at *p* = 0.05. All data are presented as mean of eight plot replicates over three individual sites ± standard error (SE). (**b**) Seed yield of *psNTP9* events and parental line trialed in 2017. The field trials were implemented during the counter-season seed increase at three locations in Puerto Rico. Seed yield performance and analysis of *psNTP9* events at each of the individual sites can be found from supplemental data. Combined site analysis of seed yield over the three trials determined 3.91 bushels per acre as LSD_0.05_ value for mean separations. Asterisk symbol (*) next to yield delta (%) values inside the figure indicates significant yield difference from parental line WM 82 at *p* = 0.05. All data are presented as mean of fifteen plot replicates over three individual sites ± SE. (**c**) Seed yield of *psNTP9* events and parental line trialed in 2018. The field trials were conducted at three different production regions in the US—Lawrence, Kentucky; Smithville, Missouri and Troy, Ohio. Seed yield performance and analysis of *psNTP9* events at each of the individual sites can be found from supplemental data. Combined site analysis of seed yield over the three trials determined 3.30 bushels per acre as LSD_0.05_ value for mean separations. All data are presented as mean of twelve plot replicates over three individual sites ± SE. (**d**) Seed yield of *psNTP9* and sibling nulls trialed in 2018. The field trials were conducted at three different production regions in the US—Lawrence, Kentucky; Smithville, Missouri and Troy, Ohio. Combined site analysis of seed yield over the three trials determined 3.30 bushels per acre as LSD_0.05_ value for mean separations. Asterisk symbol (*) next to yield delta (%) inside the figure indicates significant yield difference from corresponding sibling null at *p* = 0.05. All data are presented as mean of twelve plot replicates over three individual sites ± SE.

Figure 2 showed yield performance of *psNTP9* transgenic events 14A, 16C, and 17B trialed at multiple locations in the US over multiple years. In 2016, soybean field trials were conducted at three different locations of the primary soybean production regions—El Paso, Illinois; Henderson, Kentucky; and Atlanta, Indiana (Fig. 2a). Combined site analyses indicated that as compared to the parental line Williams 82, *psNTP9* event 17B significantly increased soybean yield by 13% (*p* = 0.05); transgenic event 14A by 9% (*p* = 0.05). In 2017, field tests were implemented during the counter-season seed increase at three locations in Puerto Rico (Fig. 2b). As compared with Williams 82, all three events showed a significant yield increase of 28–44%. In 2018, trials were conducted at three different production regions in the US—Lawrence, Kentucky; Smithville, Missouri and Troy, Ohio; sibling null segregants of each transgenic event which went through the transformation processes, but did not contain *psNTP9* gene, were included, in addition to non-transformant parent Williams 82. All three transgenic events showed yield increases of 7–13% when compared to Williams 82 (Fig. 2c), similar to results obtained from the prior two years. When compared to corresponding nulls, *psNTP9* event 17B had higher yields than the 17B null plants by 12% (*p* = 0.05) and event 14A had a 9% increased yield over the 14A null (*p* = 0.05), though 16C showed no yield difference from 16C null (Fig. 2d). Multi-year field trials in the main soybean production regions indicated that lead event 17B showed significant yield efficacy by 13% and 12% over parent Williams 82 and its sibling null, respectively.

### Seed yield increases in elite varieties harboring the introgression of *psNTP9* event 17B

**Figure 3:**
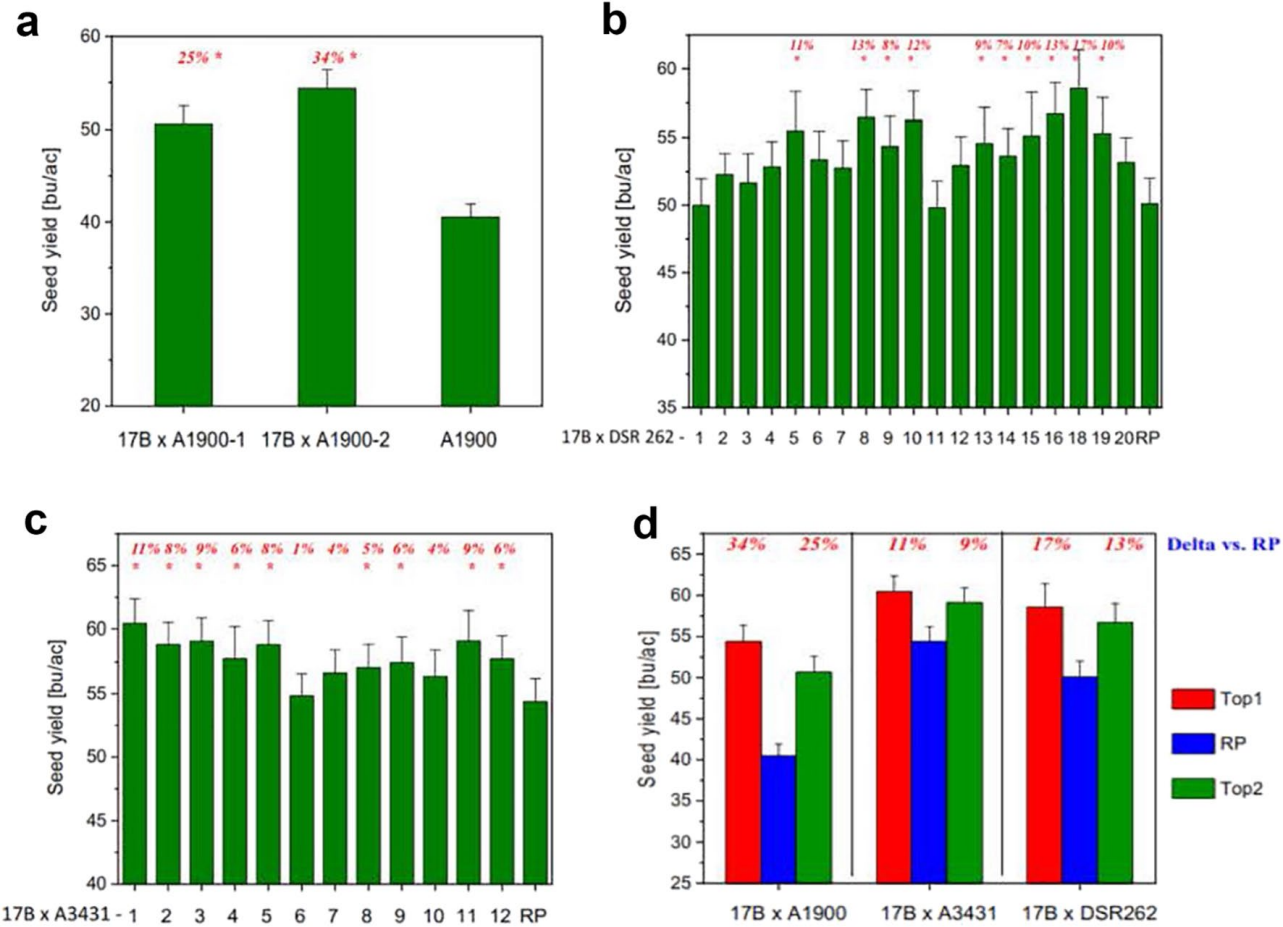
Seed yield of introgression lines of *psNTP9* event 17B into different elite varieties trialed in 2019. (**a**) 17B × A1900. Nine field trials were conducted at two locations in Argentina (Ferre, Buenos Aires; Venado Tuerto, Santa Fe) and eight locations in the US (Beatrice, Nebraska; Creston, Iowa; Macomb, Illinois; Monmouth, Illinois; Shipman, Illinois; Remington, Indiana; Tipton, Indiana; and Delphos, Ohio). Yield performance and analysis of introgression lines and recurrent parent (RP) at each of the individual sites can be found in supplemental data. Combined site analysis of seed yield over the nine trials determined 2.51 bushels per acre as LSD_0.05_ value for mean separations. Asterisk symbol (*) next to yield delta (%) inside the figure indicates significant yield difference from recurrent parent A1900 at *p* = 0.05. All data are presented as mean of 46–55 plot replicates over ten individual sites ± SE. (**b**) 17B × DSR262. Twelve field trials were conducted at three locations in Argentina (Ferre site A and B, Buenos Aires; Venado Tuerto, Santa Fe) and nine locations in the USA (Beatrice, Nebraska; Creston, Iowa; Davenport, Iowa; Macomb, Illinois; Monmouth, Illinois; Shipman, Illinois; Remington, Indiana; Tipton, Indiana; and Delphos, Ohio). Combined site analysis of seed yield over the twelve trials determined 3.36 bushels per acre as LSD_0.05_ value for mean separations. Asterisk symbol (*) next to yield delta (%) inside the figure indicates significant yield difference from recurrent parent DSR262 at *p* = 0.05. All data are presented as mean of 22–57 plot replicates over 12 individual sites ± SE. (**c**) 17B × A3431. Thirteen field trials were conducted at four locations in Argentina (Ferre site A and B, Buenos Aires; Venado Tuerto site A and B, Santa Fe) and nine locations in the USA (Beatrice, Nebraska; Creston, Iowa; Davenport, Iowa; Macomb, Illinois; Monmouth, Illinois; Shipman, Illinois; Remington, Indiana; Tipton, Indiana; and Delphos, Ohio). Combined site analysis of seed yield over the thirteen trials determined 2.64 bushels per acre as LSD_0.05_ value for mean separations. Asterisk symbol (*) next to yield delta (%) inside the figure indicates significant yield difference from recurrent parent A3431 at *p* = 0.05. All data are presented as mean of 42–55 plot replicates over 13 individual sites ± SE. (**d**) Top two performers of every introgression. Thirteen field trials were conducted at four locations in Argentina (Ferre site A and B, Buenos Aires; Venado Tuerto site A and B, Santa Fe) and nine locations in the USA (Beatrice, Nebraska; Creston, Iowa; Davenport, Iowa; Macomb, Illinois; Monmouth, Illinois; Shipman, Illinois; Remington, Indiana; Tipton, Indiana; and Delphos, Ohio). Each of yield deltas (%) above introgression line yield bars reached statistical significance at the level of *p* = 0.05, as compared to corresponding recurrent parent (RP). All data are presented as mean of 22–55 plot replicates over 9–13 individual sites ± SE.

To test whether *psNTP9* gene efficacy can be transferred into different genetic backgrounds as well as to further integrate apyrase control traits (ACT) into elite varieties for commercial development, three soybean cultivars differing in maturity groups were used as recurrent parents (RPs) for introgression of lead event 17B through multiple generations of backcrossing. Field tests of introgression lines were conducted at multiple locations of primary soybean production regions both in Argentina and in the US over 2019 crop season. Figure 3a demonstrated yield results of all introgression lines of 17B × A1900. As compared to recurrent parent A1900 with relative maturity of 1.90, both introgression line 1 and 2 had significant yield increases of 25% and 34%, respectively, at the level of *p* = 0.05. Figure 3b shows yield performance of every introgression line of 17B × DSR262 (relative maturity 2.6). Out of all 20 introgression lines, 18 lines showed increased yields, ranging from 4 to 17%, as compared to the recurrent parent. Yield efficacy from 10 introgression lines reached the significance level of *p* = 0.05, and averaged 11% yield increase with the range of 8%—17%. Each of the introgression lines of 17B × A3431 had higher yields than recurrent parent A3431 (relative maturity 3.4), across 13 locations in both countries (Fig. 3c). Out of the 12 introgression lines, 9 lines showed significant yield increases (*p* = 0.05) averaging 8%. In practice, trait integration for further commercial development is generally concentrated on the top introgression lines of targeted backcrosses. As seen in Fig. 3d, each of the top two introgression lines deriving from ACT integrated backcrosses, demonstrated significant yield benefit over corresponding RPs, with the range from 9 to 34% (*p* = 0.05). On the average, yield increase of the two top lines over corresponding RP averaged 30%, 15%, and 10% in backcrosses of 17B × A1900, 17B × DSR262, and 17B × A3431, respectively.

### Effects of expressing *psNTP9* on other agronomic attributes

**Figure 4:**
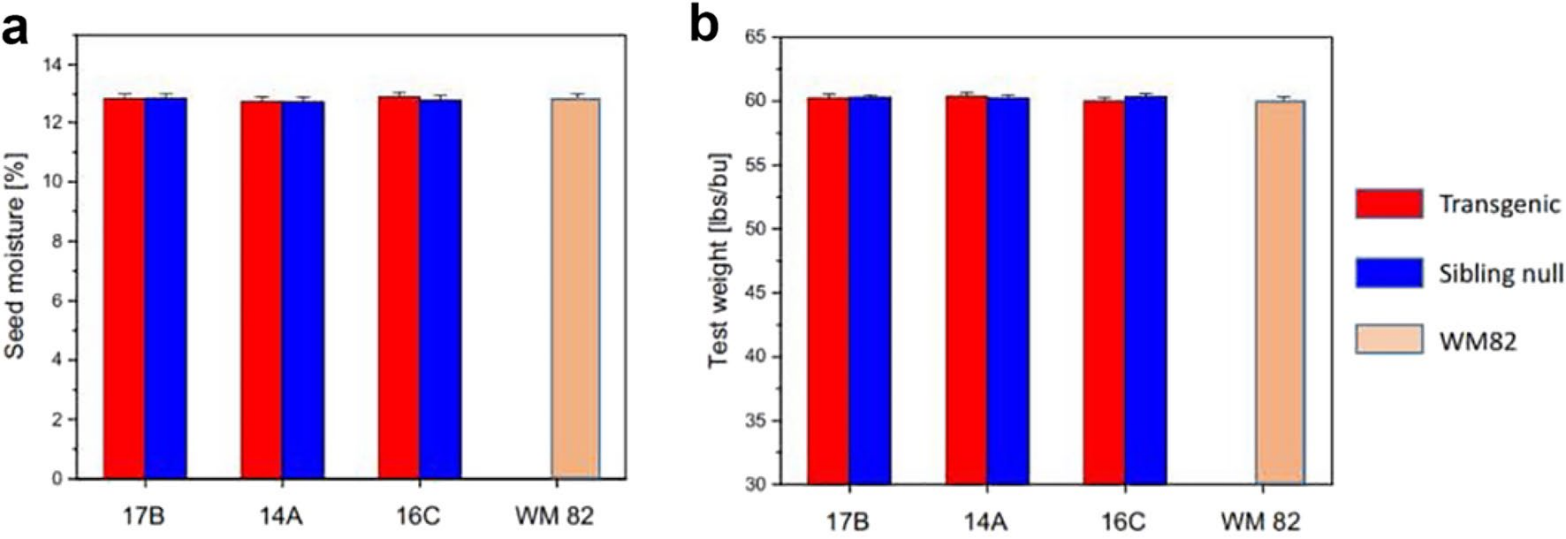
Effects of *psNTP9* gene on soybean seed moisture and test weight at harvest. (**a**) Seed moisture. Data were collected from 16 field trials including three US trials in 2018 (Lawrence, Kentucky; Smithville, Missouri and Troy, Ohio), four Argentinian trials in 2019 (Ferre site A and B, Buenos Aires; Venado Tuerto site A, Santa Fe), and nine US field trials in 2019 (Beatrice, Nebraska; Creston, Iowa; Davenport, Iowa; Macomb, Illinois; Monmouth, Illinois; Shipman, Illinois; Remington, Indiana; Tipton, Indiana; and Delphos, Ohio). Combined site analysis of transformed data of soybean seed moisture over 16 trials determined 0.15% as LSD_0.05_ value for mean separations. All data are presented as mean of 66–71 plot replicates over 16 individual sites ± SE. (**b**) Test weight**.** Data were assayed from three US trials in 2019 (Beatrice, Nebraska; Davenport, Iowa; and Macomb, Illinois). Combined site analysis of soybean test weight over three trials determined 0.44 pound per bushel as LSD_0.05_ value for mean separations. All data are presented as mean of 12–15 plot replicates over three individual sites ± SE.

During the course of plant growth and development, thorough field surveys were made to determine whether overexpression of *psNTP9* gene significantly impacts other agronomic attributes, apart from soybean seed yield. As summarized in Table 1, there were no significant differences between *psNTP9* event lines and their sibling nulls in most of plant growth and phenological attributes, such as early growth vigor score, plant stand, days from sowing to initial flowering, and days of crop cycle. Test weight and seed moisture at harvest are important economic parameters for soybean production and sales. Figure 4a shows seed moisture at harvest of *psNTP9* events against sibling nulls and parent line Williams 82, across 16 field trials in Argentina and US over 2018–2019. Figure 4b shows seed test weight assayed from three US trials in 2019. No significant differences among genotypes were found either in seed moisture or test weight. This suggests that ectopic expression of the *psNTP9* gene in soybean may not affect other agronomic attributes.

**Table 1:** Effects of *psNTP9* gene on other agronomic attributes. Early vigor score, initial and final plant stand counts, days to initial flowering, days from R1 to R8 stages, and days of crop cycle were observed from four field trials conducted in Argentina (Ferre site A and B, Buenos Aires; Venado Tuerto site A, Santa Fe) in 2019. Lodging score, seed shattering score, and performance score were surveyed prior to harvest from eight field trials in the USA (Beatrice, Nebraska; Creston, Iowa; Macomb, Illinois; Monmouth, Illinois; Shipman, Illinois; Remington, Indiana; Tipton, Indiana; and Delphos, Ohio) in 2019. Different red letters indicate that mean values for a parameter in a given row differ from one another with a *p* value < 0.05.

Agronomic parameters	Number of trials	PS events, nulls and wild type							Significance	
		14A	14A null	16C	16C null	17B	17B null	WM 82	Prob	*LSD*_0.05_^b^
Early vigor score^a^	4	3.9	3.6	3.7	3.4	4.0	3.7	3.7	0.90	0.49
Early stand count in 6 ft	4	27.7 abc	26.3 bc	28.9 ab	25.7 c	29.0 a	27.2 abc	27.9 abc	0.12	2.5
Days from sowing to initial flowering	4	95 ab	95 b	95 ab	95 b	96 a	96 a	95 ab	0.03	0.8
Lodging score^a^	8	1	1	1	1	1	1	1	1.00	n.a
Shattering score^a^	8	1	1	1	1	1	1	1	1.00	n.a
Pre-harvest performance score^a^	1	1	1	1.25	1	1	1.25	1	0.59	0.41
Final stand count in 6 ft	4	23.1 bc	22.6 c	25.1 ab	21.7 c	25.2 a	23.8 abc	23.8 abc	0.01	1.9
Days from R1 to R8	4	63	64	64	65	64	65	65	0.21	1.2
Seed moisture (%)	16	12.9	12.9	12.8	12.8	12.9	12.8	12.9	0.88	0.15
Test weight (lbs/bu)	3	60.3	60.3	60.4	60.3	60.0	60.4	60.0	0.59	0.44
Days of crop cycle	4	158 c	159 bc	159 bc	159 bc	159 b	160 a	160 ab	< 0.01	0.8

^a^Scale of scoring for vigor, plot performance, lodging and shattering: 1–5. 1 = best for vigor or plot performance; very minor lodging or seed shattering, respectively. 5 = worst for vigor or performance; very severe lodging or seed shattering, respectively.

^b^According to LSD_0.05_ value, means of PS events, nulls and wild type (WM82) for a single agronomic parameter (within the same row), when indicated with the same letter, are not significantly different at the level of p < 0.05.

### Transcript and protein expression of psNTP9 in three soybean lines

**Figure 5:**
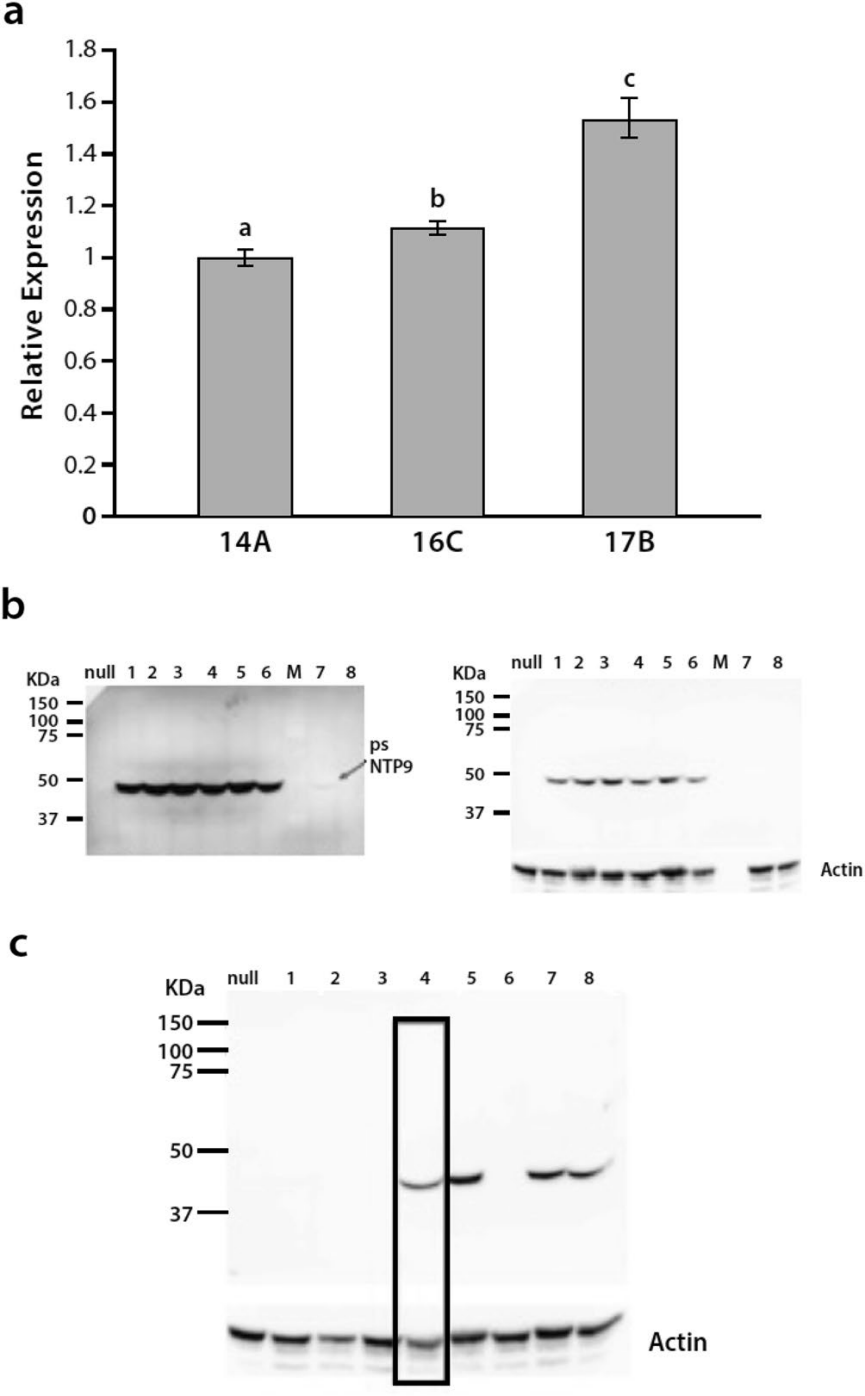
Transcript and protein abundance of psNTP9 of transgenic soybean lines. Transcript (**a**) and protein (**b**,**c**) abundance of psNTP9 is higher in leaves of the 17B line than in the 14A line of soybeans. The leaves were harvested from mature plants in the 2020 field trials in Shipman, IN. In panel (**a**) the relative expression levels were normalized to the expression level in 14A, taken as 1.0. Data represent means ± SE (n = 3). Different letters above the bars indicate statistically significant differences between the wild type and the transgenic lines using one-way ANOVA (*p* ≤ 0.01) with Tukey HSD test. In panel (**b**) equal loading was confirmed by dual staining with anti-actin antibodies. In panel (**c**) the lane outlined had less protein loaded than the following four lanes, as indicated by lower actin levels. 17B/DSR262 has a slightly higher level of psNTP9 immunostaining than 16C/A2835 and 16C/A2835, but all three have higher levels than line 14A/3431, which is below detection limit in this panel. In panel (**b**) the samples loaded were from the following events: 17B/AA3431 (lanes 1, 2, 3 and 6); 17B/D5R262 (lanes 4 and 5); homozygous line of 14A (lane 7); 14A/A3431 (lane 8). In panel (**c**) the samples loaded were from the following events: homozygous line of 14A (lane 1); 14A/3431 (lane2); null line (lane 3); 17B/A3431 (lane 4); 17B/D5R262 (lane 5); null line (lane 6); 16C/A2835 (lane 7); 16C/A2835 (lane 8). Original immunostained membranes for (**b**) and (**c**) did not detect bands below 25 kDa in any transgenic line, so images do not show this region of the blot (see Supplementary information).

As judged by qRT-PCR, leaves of 14A, 16C, and 17B harvested from the Shipman, IN field trial in 2020, all 3 transgenic lines expressed *psNTP9* transcripts, and the transcript abundance of *psNTP9* in 17B was significantly higher than in 14A (Fig. 5a; Supplementary Fig. S1A). In parallel, there was also a significantly higher psNTP9 protein content in 17B over 14A in this field trial (Fig. 5b). The levels of *psNTP9* transcripts and psNTP9 protein were only slightly higher in 17B than in 16C (Fig. 5a,c). In this comparison of psNTP9 levels, equal protein loading in each lane was confirmed by actin immunostaining (Fig. 5b,c). When less extract from a 17B line was loaded onto the SDS-PAGE gel (lane boxed in), there was less psNTP9 detected. The primary antibody used for these immunoblots was a highly specific monoclonal antibody (mAb), 8B6, which immuno-labeled only the 48 kDa psNTP9 in crude extracts of the leaves, and did not label any bands in extracts from WT samples or null transformants (Fig. 5b,c). The immunostaining in Fig. 5 and in following Figures did not detect bands below 25 kDa in any transgenic line, and membrane images do not show this region of the blot.

In leaves harvested from other field trials, RT-PCR assays of *psNTP9* confirmed its expression in all three transgenic lines (Supplementary Fig. S1A), and in immunoblots of leaf, flower and root tissues harvested from greenhouse-grown soybeans, the psNTP9 protein was detected in immunoblots of all three tissues of 17B by the 8B6 mAb (Supplementary Fig. S1B). The blot also shows that psNTP9 was expressed in the flower and root tissues of the 14A transgenic line and in the flower tissue of the 16C transgenic line (Supplementary Fig. S1B). The minimal level of purified psNTP9 detectable in immunoblots using mAb 8B6 as the primary antibody is approximately 0.1 ng (Supplementary Fig. S2).

### Leaf transcriptome analyses

Based upon early data with greenhouse grown plants, prior to collection and analyses of data from field-grown plants, leaf tissues from WT and transgenic line 14A plants were selected to transcriptome analyses. Compared with WT, 996 genes were differentially-expressed (DE) in 14A leaf tissues and 753 genes were up-regulated. Fold-change expression values for selected genes were verified by qRT-PCR analyses (Supplementary Table S2). Enriched GO BioProcess terms associated with DE genes (DEG) are shown in Table 2. Genes involved in RNA processing, ribosome biogenesis and protein synthesis comprised approximately 81% of up-regulated DEGs. Genes associated with fatty acid and wax biosynthesis and cuticle development were also over-represented in this category. Enriched down-regulated categories included regulation of cell division, DNA replication and transcription. Expression of genes regulating epidermal cell fate specification and stomatal complex development was also decreased.

**Table 2:** Enriched GO BioProcess terms associated with DEG in soybean 14A leaf tissues. All terms are statistically overrepresented by ≥ fivefold (FDR-corrected *p* ≤ 0.05).

GO ID	GO BioProcess description	Genome GO count	Expressed GO count	Expected GO count	Fold over-represented
**Up-regulated genes (753)**					
**Mitochondrial protein import**					
GO:0045039	Protein import into mitochondrial inner membrane	19	6	0.350	17.2
GO:0006626	Protein targeting to mitochondrion	235	22	4.327	5.1
**Fatty acid and wax biosynthesis, cuticle development**					
GO:0000036	ACP phosphopantetheine attachment site binding involved in fatty acid biosynthetic process	29	8	0.440	18.2
GO:0010025	Wax biosynthetic process	59	7	1.086	6.4
GO:0042335	Cuticle development	131	12	2.412	5.0
**Protein synthesis**					
GO:0006412	Translation	763	261	14.048	18.6
GO:0006414	Translational elongation	81	23	1.491	15.4
**RNA processing, ribosome biogenesis**					
GO:0030515	snoRNA binding	8	5	0.121	41.2
GO:0042274	Ribosomal small subunit biogenesis	19	14	0.350	40.0
GO:0008097	5S rRNA binding	11	5	0.167	30.0
GO:0003735	Structural constituent of ribosome	733	271	11.114	24.4
GO:0042254	Ribosome biogenesis	264	103	4.861	21.2
GO:0000462	Maturation of SSU-rRNA from tricistronic rRNA transcript (SSU-rRNA, 5.8S rRNA, LSU-rRNA)	13	5	0.239	20.9
GO:0001510	RNA methylation	418	159	7.696	20.7
GO:0006407	rRNA export from nucleus	11	4	0.203	19.8
GO:0019843	rRNA binding	44	7	0.667	10.5
**Down-regulated genes (243)**					
**Regulation of cell division**					
GO:0042127	Regulation of cell proliferation	108	14	1.240	11.3
GO:0010389	Regulation of G2/M transition of mitotic cell cycle	155	15	1.780	8.4
GO:0000910	Cytokinesis	92	7	1.056	6.6
GO:0000911	Cytokinesis by cell plate formation	471	28	5.409	5.2
**Microtubule motor activity, cytoskeleton organization**					
GO:0003777	Microtubule motor activity	184	25	1.522	16.4
GO:0007018	Microtubule-based movement	184	21	2.113	9.9
GO:0051225	Spindle assembly	98	8	1.125	7.1
**Epidermal cell, stomatal complex development**					
GO:0010374	Stomatal complex development	44	7	0.505	13.9
GO:0009957	Epidermal cell fate specification	33	5	0.379	13.2
**Epigenetic regulation of gene expression**					
GO:0016458	Gene silencing	156	15	1.791	8.4
GO:0016572	Histone phosphorylation	159	12	1.826	6.6
GO:0034968	Histone lysine methylation	258	15	2.963	5.1
**Regulation of DNA replication and transcription**					
GO:0006275	Regulation of DNA replication	255	20	2.928	6.8
GO:0006270	DNA replication initiation	159	11	1.826	6.0

### Nitrogen assimilation genes are up-regulated in 14A leaves

Although nitrogen assimilation genes were not enriched in 14A leaves, several genes encoding the nitrate transporter NITR2;1 (*Glyma.17G096600*), nitrate reductase 1 (NIR1; *Glyma.07G212800*, *Glyma.02G132100*) and glutamate synthase 1 (NADH) (GLT1; *Glyma.06G127400*. *Glyma.04G236900*) were upregulated 1.5 to two-fold. NTR2;1 mediates high-affinity uptake of nitrate into the chloroplast^31^. In the chloroplast, NIR1 reduces nitrate to ammonia^32^ and GLT1 (GS-GOGAT) catalyzes a reaction that assimilates non-photorespiratory ammonia into glutamate and general nitrogen (N) metabolism^33^.

### Genes related to cuticle formation are differentially-expressed in 14A leaves

Soybean genes functioning in the synthesis of very long-chain fatty acids (VLCFA), cutin monomers and cuticular waxes, or regulation of cuticle development, were identified using Soybase genome annotation data, which includes Arabidopsis ortholog information based on top BlastP hits (TAIR10), and GO BioProcess terms related to cuticle development. Most enzymes, carrier proteins and transporters involved in these processes are encoded by gene families in Arabidopsis. Supplementary Table S3 summarizes the normalized expression of DE soybean orthologs of Arabidopsis genes involved in cuticle formation. Expression of non-DE orthologs for each gene are also included to provide a more complete understanding of how DE genes may contribute to changes in cuticle formation and properties.

All DEG encoding enzymes involved in synthesis of fatty acid precursors of cutin and cuticular waxes (see Refs.^34,35^) were up-regulated in 14A leaves. These included acyl carrier proteins ACP1 and ACP4, which serve as co-factors in fatty acid synthase (FAS) complex reactions in the plastid, enoyl-ACP reductase (ENR1), which catalyzes the final reduction step in each FAS cycle (2-C addition), and fatty acyl-ACP thioesterase B (FATB), which terminates the elongation cycle by hydrolyzing ACP, releasing free C16/C18 fatty acids. Up-regulation of long-chain acyl-CoA synthetase 1 (LACS1), which activates the free fatty acids to CoA esters, and cytosolic acyl-CoA-binding protein 6 (ACBP6), which facilitates acyl-CoA export from the plastid to the endoplasmic reticulum site of VLCFA, cutin and wax biosynthesis, was also seen. Genes important for VLCFA synthesis included cytosolic ATP citrate lyase subunit B2 (ACLB-2), which generates acetyl-CoA used in the synthesis of malonyl CoA, the 2-C donor for VLCFA elongation, and nine genes encoding all four enzymatic activities in the ER fatty acid elongase (FAE) complex. Up-regulation of the single soybean gene *ECERIFERUM 2 (CER2)*, encoding an additional component of the FAE that facilitates elongation of fatty acids greater than 28-C^36^ and provides VLC acyl-CoA for wax biosynthesis, was also noted. In most cases, DE genes were among the most highly-expressed paralogs encoding each enzyme activity.

Relatively few soybean genes involved in wax synthesis^35^ were DE in 14A leaves, but several changes are noteworthy. Of the nine paralogs of VLCFA CoA reductase (*CER4*^37^), down-regulation of the highly-expressed *Glyma.11g185100* may result in decreased flux through the alcohol-forming branch of wax synthesis. However, none of the three highly-expressed *WSD1* genes encoding the bifunctional wax ester synthase/diacylglycerolacyltransferase that catalyzes the second step of this pathway^38^ were DE in 14A leaves. In contrast, up-regulation of three highly-expressed VLC aldehyde decarbonylase complex genes^39^ (*CER1*, *Glyma.03G101800*; *CER3*, *Glyma.13G091200* and *Glyma.17G069100*) support increased formation of VLC-alkanes, despite decreased expression of *Glyma.03G101200* and *Glyma.07G114200*. CER3 expression in 14A leaves increased 4.4-fold, relative to WT leaves, in qRT-PCR assays, independently confirming the up-regulation of this gene (Supplementary Table S2). Three soybean genes encoding the midchain alkane hydroxylase 1 (MAH1), which catalyzes formation of VLC-secondary alcohols and VLC-ketones in latter steps of the alkane pathway^40^, were not differentially expressed (data not shown).

Synthesis of cutin monomers occurs in the ER via ω-omega-hydroxylation (CYP86 subfamily of cytochrome P450s) and midchain hydroxylation (CYP77 subfamily) of C16/C18-CoA precursors, followed by glycerol-3-phosphate acyltransferase (GPAT) formation of 2-monoacylglycerols^35^. In soybean leaves, genes encoding CPY86 and CYP77 hydroxylases were not DE, but expression of a soybean GPAT8 (*Glyma.03G008300*), which belongs to the cutin-associated clade GPAT4,6,8^41^, increased 1.5-fold. A second GPAT8 ortholog (*Glyma.07G069700*) was expressed at tenfold higher levels and was not DE in 14A leaves. Cutin synthase catalyzes the final step in cutin synthesis, polymerization of cutin monomers, forming the structural polyester of the cuticle^42^. Two cutin synthases have been functionally characterized (tomato DD1, whose closest Arabidopsis ortholog is *CUS1/LTL1* (AT3G04290)^42^ and Arabidopsis *CUS2*^43^. In soybean 14A leaves, the two *CUS1* genes (data not shown) and six highly-expressed *CUS2* genes were not DE.

Transport of cutin monomers and cuticular waxes from the ER to the epidermal surface is poorly understood^35^. Homodimers of ABCG11 half-transporters and heterodimers of ABCG11/ABCG12 function in secretion of cuticular lipids in epidermal cells of Arabidopsis stems^44^. Soybean ABCG11 genes were not DE, including the highly-expressed *Glyma.09g160000* and *Glyma.16g209400* genes. Soybean genes annotated as ABCG12 were not identified but BlastP searches using the Arabidopsis ABCG12 (AT1G51500) sequence identified 10 highly-similar ABCG15 (CER5) orthologs. None were DE except for *Glyma.03g135525*, which exhibited approximately 20-fold lower expression than the two most highly-expressed genes in this family (Supplementary Table S3). Three soybean genes encoding apoplastic GPI-anchored lipid transfer proteins (LTPG1, LTPG2) were up-regulated in 14A leaves. Both proteins function in cuticular wax export and accumulation^45^. Expression of two cell wall-localized members of a large family of non-specific lipid transfer proteins, LTP1 and LTP5, was increased > twofold in 14A leaves but the role of LTP in cuticle formation is unclear^46^.

Transcriptional and post-transcriptional mechanisms regulating cuticle formation have been previously reviewed^35,47^. In 14A leaves, three MYB94 genes were up-regulated. Lee et al.^48^ reported that MYB94 and functionally-redundant MYB96 activate a number of genes important for synthesis of VLCFA (*CER6, KCR1*) and cuticular waxes (*CER1, CER3, WSD1*), consistent with findings in the present study.

### Genes that regulate stomatal development are downregulated in 14A leaves

Among the 243 downregulated genes, seven are involved in stomatal complex development and five function in epidermal cell fate specification (Supplementary Table S4). bHLH transcription factors SPEECHLESS (SPCH) and MUTE help regulate the differentiation of protodermal cells into mature stomata^49^. Expression of three *SPCH* and two *MUTE* orthologs was down-regulated 1.5- to 2-fold in 14A leaves (Supplementary Table 4). Interestingly, a number of genes encoding a signaling peptide and components of a receptor-mediated signaling cascade that negatively regulates SPCH activity were also down-regulated, perhaps as compensatory response to decreased *SPCH* expression.

### Increased chlorophyll and protein content of 14A and 17B leaves

**Figure 6:**
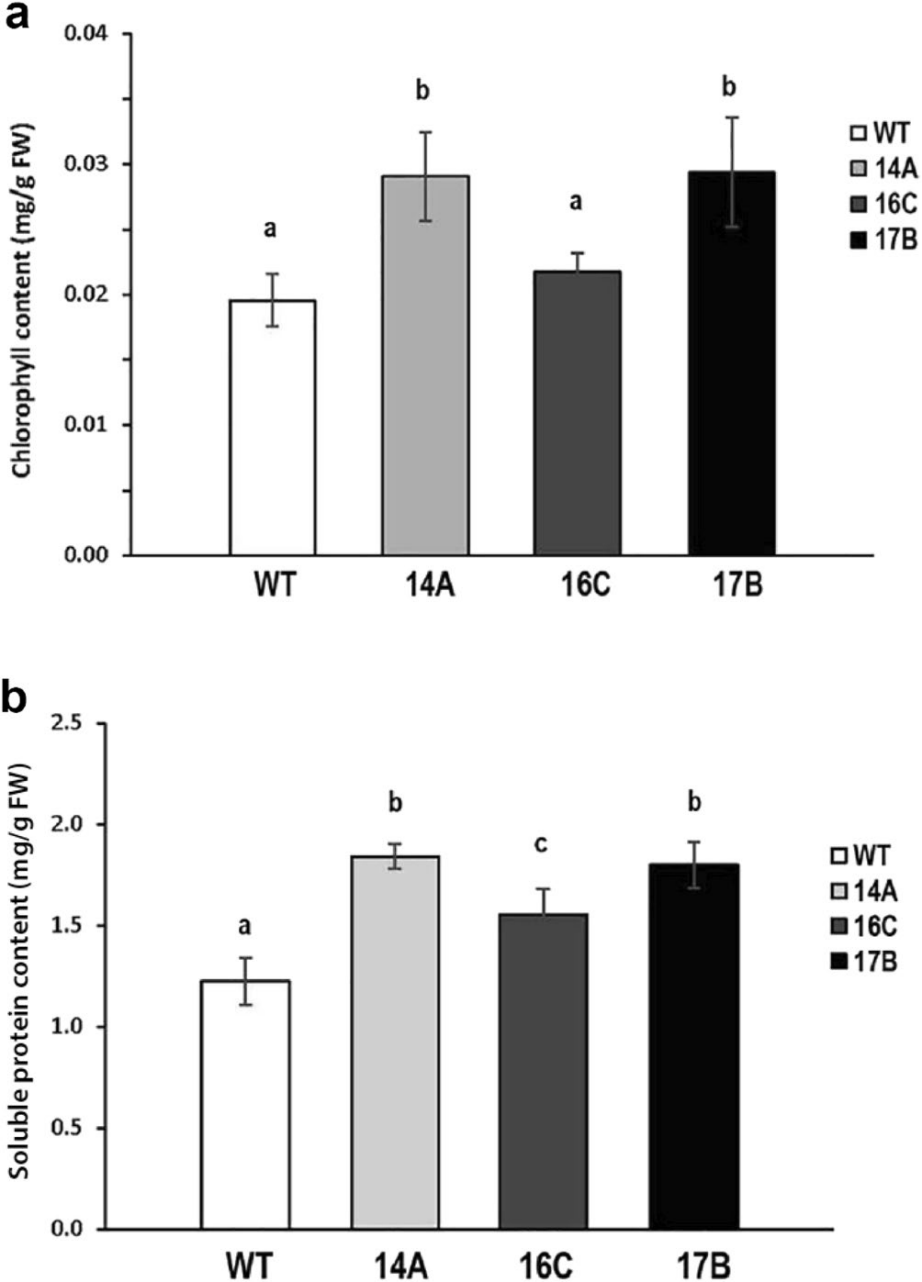
Leaves from the transgenic lines 14A and 17B have higher contents of total chlorophyll and soluble protein. (**a**) Leaves of 14A and 17B transgenic events have higher total chlorophyll contents than WT leaves. (**b**) The total soluble protein contents of 14A, 16C and 17B transgenic leaves from thirty-four-day-old greenhouse grown plants at V3 stage (the third trifoliate stage) is higher than in WT leaves. The data shown are means ± SE from 3 biological replicates (*n* = 6). Different letters above the bars indicate statistically significant differences between the wild type and the transgenic lines using one-way ANOVA (*p* ≤ 0.01) with Tukey HSD test.

Total chlorophyll and protein contents were measured from completely opened V3 stage leaves of wild type and transgenic plants. The chlorophyll content of 16C leaves was not significantly different than in leaves from wild type plants. There was 40% increase in the chlorophyll content of both 14A and 17B transgenic leaves when compared to wild type leaves (Fig. 6a). There was also a 40% increase the total soluble protein content in 14A and 17B leaves and a smaller but significant 20% increase in 16C leaves, compared to wild type leaves (Fig. 6b).

### Increased thickness of the cuticle and cell wall in 17B leaves

**Figure 7:**
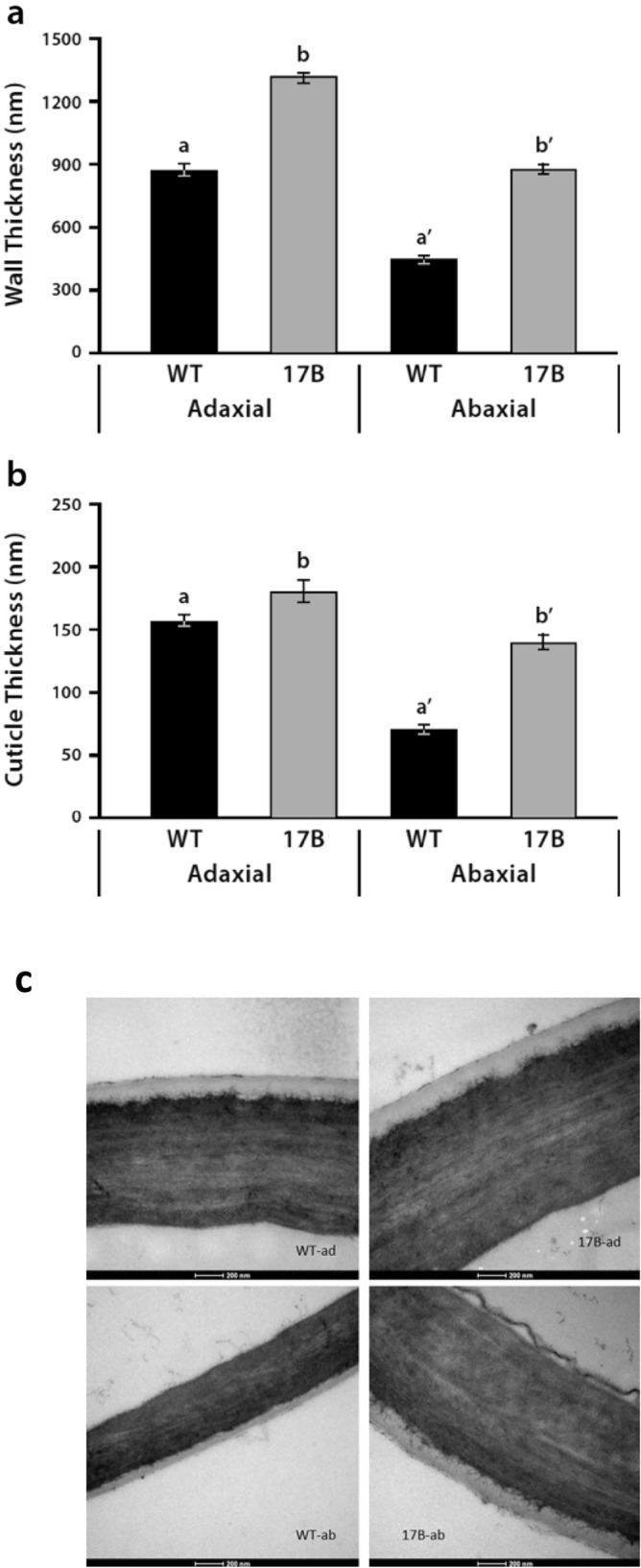
Leaves from the 17B transgenic soybean line displays a significant increase in cuticle and wall thickness of epidermal cells compared to wild-type (WT). Analyses of transmission electron microscopy (TEM) images of WT and 17B leaves show (**a**) an increase in the adaxial and abaxial thickness of the 17B epidermal cell walls compared to WT (adaxial *p* < 0.001, abaxial *p* < 0.001) and (**b**), increase in adaxial and abaxial cuticle thickness of 17B epidermal cells compared to WT (adaxial *p* < 0.05, abaxial *p* < 0.001). Data represent means ± SE, based on the Student’s *t*-test. Representative TEM images (**c**) of adaxial and abaxial epidermal cells from WT and 17B leaves.

Cuticle and wall thickness of epidermal cells in WT and 17B transgenic leaves was examined by direct measurements using TEM images. Both adaxial and abaxial cell wall and cuticle thickness of epidermal cells were greater in leaves of the transgenic 17B line compared to wild-type (Fig. 7a–c). The differences between WT and 17B leaf epidermal cells for both cell wall and cuticle thickness were greatest for the abaxial side of leaves. Adaxial cuticle width was on average 14% percent greater in the 17B mutant epidermal cells compared to WT while it was 95% greater on the abaxial side of epidermal cells (Fig. 7a). Cell wall thickness showed a similar pattern with adaxial wall width in 17B epidermal cells averaging 50% greater than WT while it was 95% greater for abaxial cell walls (Fig. 7b). Because the greatest increases in cuticle and wall thickness occurred on the abaxial sides of the 17B leaves, the ratios between adaxial and abaxial cuticle and cell wall thickness were greatly reduced in mutant leaves compared to WT leaves (WT cell wall ratio = 1.94, cuticle ratio = 2.2; 17B cell wall ratio = 1.49, cuticle ratio = 1.29). These differences in cell wall and cuticle thickness can be seen in representative TEM photos (Fig. 7c).

### Reduced transpiration water loss and chlorophyll leakage in leaves of 14A and 17B

**Figure 8:**
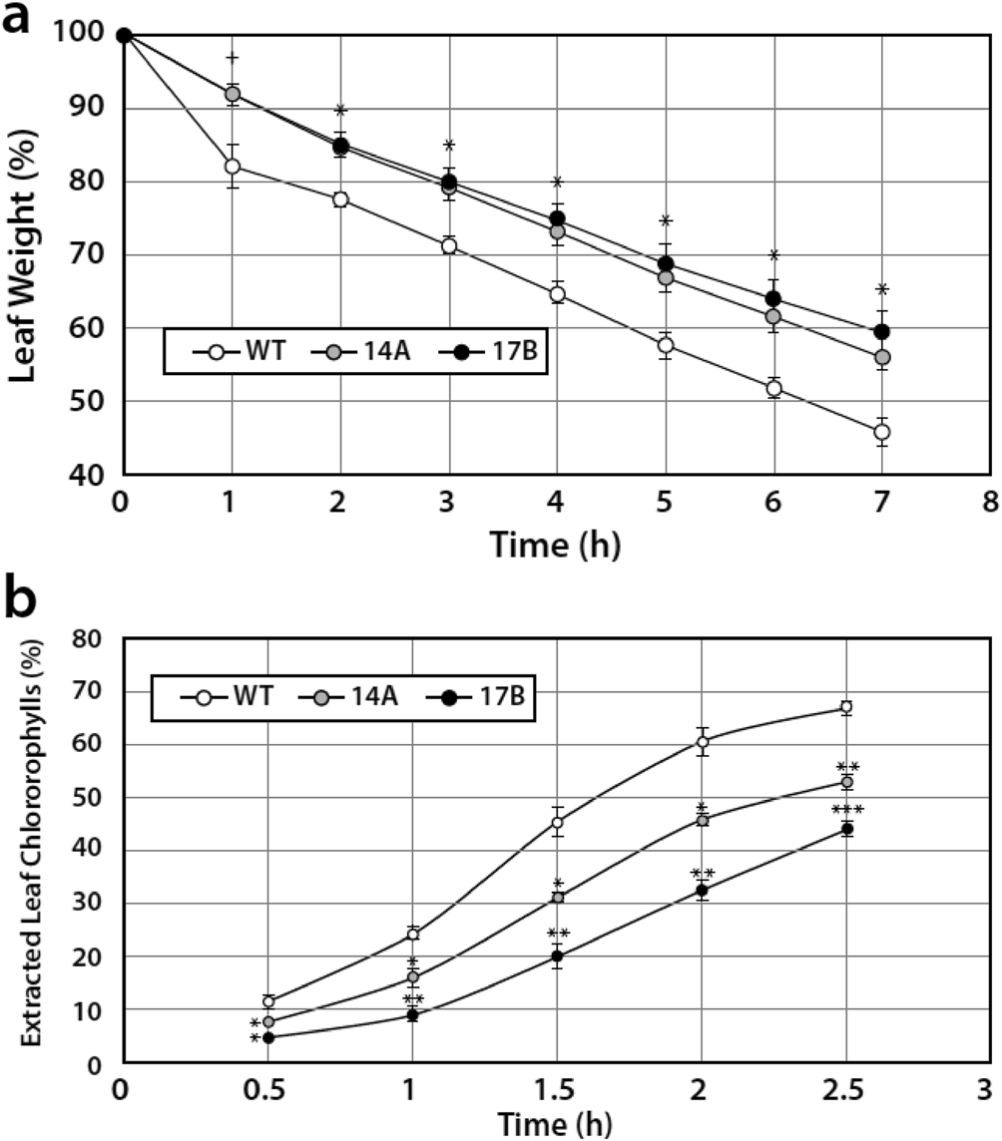
Leaves from the transgenic lines 14A and 17B show decreased cuticle permeability compared to wild-type (WT). Cuticle permeability assays using detached V3 stage leaves of WT and transgenic lines 14A and 17B. (**a**) Water loss assay (+p ≤ 0.05; *p ≤ 0.01) and (**b**) Chlorophyll leakage assay (*p ≤ 0.05; **p ≤ 0.01; ***p ≤ 0.001). Data represent means ± SE (n = 3), with statistical differences based on the Student’s *t*-test.

Assays of transpiration water loss (Fig. 8a) and chlorophyll leakage (Fig. 8b) from detached leaves were used to investigate differences in cuticle permeability between WT and 14A, 17B leaves. Both transpiration water loss and chlorophyll leakage were significantly reduced in the leaves of 14A and 17B compared to WT leaves. The leaves of the 17B transgenic line had statistically significantly longer trichomes than wild-type leaves (Supplementary Fig. S4). Longer trichomes of 17B leaves were observed on both abaxial and adaxial surfaces of the leaves. In another study related to leaf control of transpiration water loss, initial studies indicated that the leaves of the 17B transgenic line have a higher trichome density (data not shown).

### Decreased stomatal density in leaves of 14A and 17B transgenic lines

**Figure 9:**
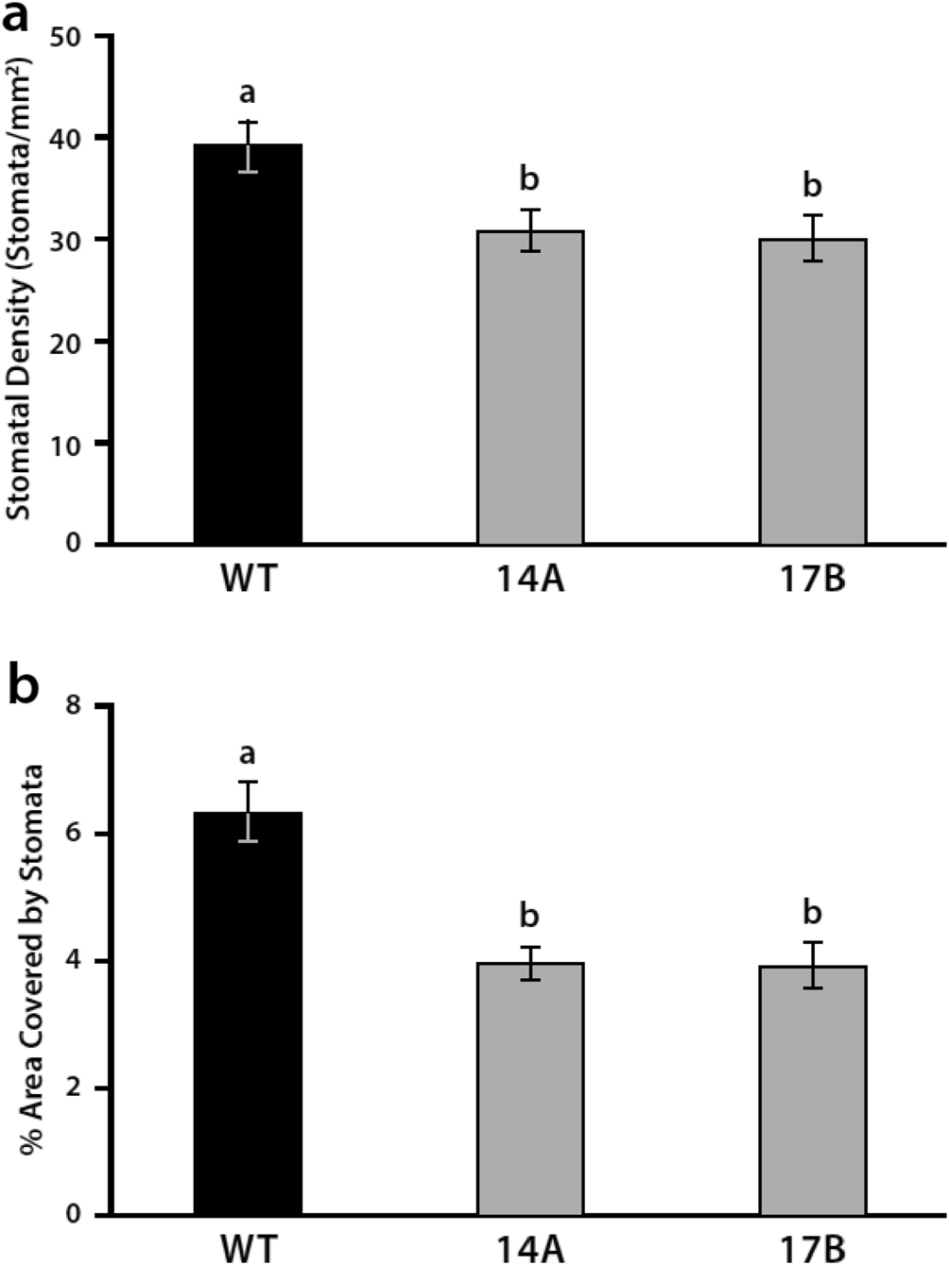
Leaves from 14A and 17B transgenic soybean lines display a significant decrease in stomatal density and stomatal coverage compared to wild-type (WT). Analysis of stomata of WT, 14A and 17B leaves shows (**a**) lower stomatal density for 14A and 17B leaves (*p* < 0.05), and (**b**) smaller percent area covered by stomata for 14A and 17B leaves compared to WT (*p* < 0.001). Data represent means ± SE, with statistical differences based on the Student’s *t*-test.

Analysis of stomatal development on abaxial side of leaves in WT and the 14A and 17B transgenic line showed statistically significantly lower average stomatal density in 14A and 17B leaves (Fig. 9a). Similarly, measurements of the peel area covered by stomata in the abaxial side of 14A and 17B leaves showed statistically significantly less stomatal coverage compared to WT (Fig. 9b).

## Discussion

In the Veerappa et al.^19^ study, the seed yields in the 14A, 16C, and 17B soybean lines were remarkably higher than WT seed yields (31%, 45%, and 54% higher, respectively), when grown under ideal greenhouse conditions. However, positive yield results observed in the greenhouse are often not replicated under the more stressful field conditions, or are observed in only a minority of field trials^50^. What is remarkable about the data presented here is that the increased seed yields reported for three different transgenic soybean lines expressing the *psNTP9* transgene were consistently observed over several growing seasons in diverse soil types and climate conditions. These increases, which typically ranged over 10% to as high as 44%, would all be considered of significant agricultural value.

Each soybean line exhibited a single insertion of the transgene and there did not appear to be positional effects on expression of neighboring genes in either 16C or 17B lines, although the insertion site in 17B appears to be within the promoter of *Glyma.03G074600*. qRT-PCR results indicate this gene does not change expression in 17B, so the insertion site is probably not a strong *cis*-acting element in this gene. In 14A, qRT-PCR analysis confirmed there was strong upregulation of *Glyma.11G183700*, even though this gene is located ~ 10 kb downstream from the insert. The GmTGA14 product of this gene is closely-related to GmTGA17, whose overexpression confers drought and salt stress tolerance in soybean seedlings^52^. Thus, increased expression of *Glyma.11G183700* in 14A may enhance abiotic and biotic stress tolerance, contributing to increased seed yield in this line. However, since this gene was not strongly up-regulated in 17B leaves, and in all four field trials both 17B and 14A events outperformed the WT line in seed yield and, in one of the four trials, all three events had higher seed yields than the WT line, the overall results of this study argue that the yield phenotypes observed in the transgenic lines were due to *psNTP9* expression rather than to the insertion site effects. In the Shipman field trial, the higher yield of 17B over 14A correlated with higher expression of *psNTP9*, but this correlation would have to be tested in many other field trials to rigorously evaluate whether yield was consistently related to the level of *psNTP9* expression.

The seed yield increases in the greenhouse and in initial field trials in 14A, 16C and 17B were all measured in comparison to the Williams 82 variety of soybean, which is no longer a commercially valuable variety^53^. A critical question was whether transferring *psNTP9* into the genetic backgrounds of elite varieties could also increase the yield of those soybeans. Although which line produced the highest yield per acre varied from field site to field site and from year to year, the 17B line more often produced the highest yield in most field studies, just as it did in greenhouse studies^19^, thus it was selected to be introgressed into elite lines. When field tests of relative yields from these introgressed events were carried out, once again events expressing *psNTP9* had significantly higher yields than the null controls. Because trait integration for further commercial development is typically concentrated on the top introgression lines of targeted backcrosses, the average yield increase of 10–30% in the top two lines of 17B introgressed into elite lines would be considered of high potential value for commercial development.

Several of the phenotypic changes observed in the transgenic lines could help account for the increased seed yields. For example, the significantly elevated chlorophyll contents of leaves of all transgenic lines indicates a higher nitrogen (N) content, which is consistent with the observed up-regulation of important N-assimilation genes in 14A plants. Similarly, the increased protein contents of transgenic leaves would be supported by increased N assimilation and the remarkable number of up-regulated genes related to protein synthesis in 14A leaves supports this as a major biosynthetic activity that differentiates transgenic lines from WT. Nitrogen is the main nutrient remobilized from senescing leaves and plays a major role in determining seed yields and quality^54^. Increased N remobilization from leaves of transgenic lines during later stages of development may help to explain increased yields in these lines. Of course, higher chlorophyll contents would also support higher rates of photosynthesis leading to higher tissue mass, which had been previously observed in the transgenic lines^19^.

A drought tolerance and increased yield phenotype in soybean plants ectopically expressing *psNTP9* was previously reported^19^. These plants exhibited both an enhanced root system architecture for water uptake and decreased transpiration water loss in detached stems and leaves. In the present study, genome-wide expression profiling of WT and 14A leaves was used to further investigate molecular mechanisms that may regulate the drought tolerance phenotype. Gene expression data support enhanced cuticle development and decreased stomatal density in 14A leaves and these phenotypes were experimentally confirmed.

The cuticle plays a major role in regulating transpiration water losses from terrestrial plant tissues^35^. Gene expression profiles suggest an increased synthesis of cuticular wax in the transgenic line 14A. Increased expression of several lipid transfer proteins in this line may increase export of waxes into epidermal cell walls^45^, increasing cuticle thickness. Crops with more cuticular wax have improved water use efficiency (WUE) and increased drought tolerance and yields than non-waxy crops^47^. Increased cuticle thickness in 17B leaves, relative to WT or 14A leaves may contribute to the observed drought tolerance of this line.

Previous studies, however, have generally found that cuticle water permeability (CWP) is poorly correlated with cuticle thickness or total amount of wax or cutin. Cuticle substructure and wax composition appear to be more important^35,55^. A high proportion of non-polar wax constituents like alkanes, rather than nonaliphatic wax compounds appears to be associated with lower CWP^35^. In Arabidopsis, changes in *CER1/CER3* expression^39,56^ or CER3 activity^57^ regulate alkane synthesis. Increased synthesis of alkanes resulted in increased cuticle and epidermal cell wall thickness and drought tolerance in this plant^57^. The coordinated down-regulation of *CER4* and up-regulation of *CER1/CER3* in 14A plants supports increased activities in the alkane-forming branch of wax synthesis. Alkanes are the major wax constituent in soybean leaves^58^ and this alkane fraction increases markedly in response to drought stress in soybean and other plants^47,58^. Thus, the predicted alkane-enriched cuticles of 14A leaves would be expected to be less permeable than those of WT leaves, and this was shown experimentally for both 14A and 17B lines. Lower cuticle permeabilities in these plants likely contribute to their improved drought tolerance^19^.

Decreased stomatal density in 14A leaves may result from decreased expression of several *SPCH* and *MUTE* transcription factor genes. The mechanisms by which this occurs are unknown. However, reduction of stomatal density (SD) via targeted changes in the genetic network that regulates stomatal development has produced plants with enhanced WUE and drought tolerance^59^. This results from reduced stomatal transpiration, which is the major contributor to leaf transpiration^60^. The combination of reduced SD and increased trichome density has been shown to improve WUE in tomato plants^61^. Reduced SD may also impact cuticular transpiration indirectly. Márquez et al.^60^ proposed that “pores” in the cuticle around stomata and trichomes are the main source of gas leakage across the cuticle. Thus, decreased SD in 17B leaves would be expected to decrease both cuticular and stomatal transpiration. Stomatal transpiration is also regulated by changes in stomatal aperture. Previous studies with Arabidopsis have shown that apyrases regulate stomatal aperture by modulating eATP levels in guard cells^62,63^ and in the previous greenhouse study we showed that expression of *psNTP9* in soybean increased the sensitivity of the stomatal response to ABA treatment^19^.

Enhanced cuticle development and decreased SD in *psNTP9* transgenic lines would result in physical barriers that may further limit insect and pathogen attack^35,64,65^, perhaps, contributing to increased seed yields in field-grown plants.

It is remarkable that the drought-tolerance phenotype is observed in greenhouse-grown transgenic lines which did not experience prolonged drought stress. Decreased cuticular transpiration (lower cuticle permeability) and stomatal transpiration (decreased stomatal density) may increase yields by enabling the plant to better adapt to actual water stress conditions in the field, which may occur intermittently during the growing season. This attribute will be even more important with climate change^66^.

Many of the trait changes observed in the transgenic lines likely resulted from the gene expression changes that were induced by the ectopic expression of *psNTP9* in these lines, and the probable links between specific gene expression changes and trait alterations were noted in the Results section. As to what molecular mechanisms could explain how *psNTP9* expression could result in transcriptomic changes, a consideration of two subcellular locales in which the protein functions, i.e., the ECM and/or the nucleus, would be instructive. In peas, the psNTP9 protein has been immunolocalized both in the ECM and in the nucleus^67^, and, in soybean extracts, it co-purifies both with isolated nuclei and with wall preparations (Supplementary Fig. S3). Its primary function in the wall would be as an ecto-NTPDase, to limit the concentration of eATP, a signaling agent that plays a major role in controlling gene expression both in animals and in plants^68^. Its function in the nucleus is as yet undefined. However, the fact that it was originally purified from a chromatin fraction of the nucleus^24^, and that it induces genome wide changes in gene expression, would suggest that its NTPDase activity or, perhaps, protein–protein interactions in the chromatin could have a major impact on transcription.

The special significance of the field trial results reported here at multiple locations in multiple years is that the yields of the transgenic events were consistently enhanced over those of the controls, and the yields of transgenic lines derived from introgressions into various elite soybean varieties were significantly higher than those of the recurrent parents. To help explain the enhanced yields, our report offers plausible mechanisms that are supported by a unique combination of genetic, physiological, anatomical, and agronomic assays.

## Methods

### Genomic DNA isolation and Southern blot for detection of T-DNA copy number

Soybean genomic DNA was isolated from the 3–6 leaves of transgenic soybean lines by a modification of the method of Dellaporta et al.^69^. Southern blot hybridization was carried out using DIG High Prime DNA Labeling and Detection Starter Kit II following the manufacturer’s instructions (Roche Applied Science). Fifteen micrograms of genomic DNA were digested with either XbaI or BamHI. Digested DNA was separated on 0.8% by electrophoresis and transferred to a positively charged nylon membrane (Amersham Hybond N). The membrane was hybridized at 42 °C with a digoxigenin-labeled *Bar* probe 42 °C generated using a PCR DIG Probe Synthesis Kit (Roche) and BarF and BarR primers specific to NOS promoter and NOS terminator sequences flanking the *BAR* gene in the T-DNA construct, respectively (Table. 1). The blot was washed with 2 × SSC, 0.1% sodium dodecyl sulfate twice for 5 min at room temperature, 0.5 × SSC, and 0.1% sodium dodecyl sulfate twice for 15 min at 68 °C. The signals were detected by anti-digoxigenin antibody and chemiluminescent substrate for alkaline phosphatase (Sigma) followed by exposure on X-ray film.

### Identification of T-DNA insertion sites via PCR-based methods

For the identification of the insertion site of the T-DNA, two PCR-based methods of thermal asymmetric interlaced polymerase chain reaction (TAIL-PCR) and inverse PCR were used. TAIL-PCR was used to determine the insertion loci of transgenic soybean by a modification of the method of Singer and Burke^70^. The PCR condition and program TAIL-PCR were carried out with three T-DNA vector-specific primers (LB 1–3) and six arbitrary degenerate primers (AD 1–6) (Supplementary Table S1). Three PCR reactions (primary, secondary and tertiary TAIL PCR) were performed. The tertiary TAIL-PCR product was cloned into the pGEM-T easy vector and sequenced by Sanger sequencing at the University of Texas Institute of Cellular and Molecular Biology core facility.

Inverse PCR was also used to identify the insertion sites of T-DNA in transgenic soybean lines. The inverse PCR method was performed referring to Rønning et al.^71^ with slight modifications. Ten micrograms of genomic DNA were digested with either BamHI or XbaI. The digested DNA was self-ligated with T4 DNA ligase. The ligated DNA fragments were used as the DNA templates for primary PCR with the primer set of LB1/Bar3R or NTP1R/NTP5F. Using the primary PCR product, secondary PCR was performed using the nested primer set of LB2/Bar2R or NTP6F/35SproR specific to the T-DNA construct. The primers used in inverse PCR are found in Supplementary Table S1. The PCR products were confirmed on the agarose gel. The confirmed secondary PCR product was sequenced by Sanger sequencing.

The sequences from TAIL PCR and inverse PCR were analyzed using Quick Wm82 Genome BLAST at SoyBase (https://www.soybase.org/) to identify integration loci in soybean chromosomes^72^.

### PCR validation of T-DNA insertions

Primers were designed to generate an amplicon that contains a small portion of 5′ T-DNA end and 5′ flanking region or 3′ T-DNA end and 3′ flanking region. Primer sets of 35SproR/Gm11R, Gm03F/LB3, and LB3/GM14R were used for the insertion loci validation of soybean transgenic lines of 14A, 17B, and 16C, respectively (Supplementary Table S1). Untransformed soybean genomic DNA and distilled water were used as negative controls in PCR reactions. PCR was performed using genomic DNA of three transgenic plants and PCR SuperMix (Invitrogen, (Thermo Fisher Scientific) according to the manufacturer’s instructions. The PCR program was as follows: 94 °C (4 min); 28 cycles of and 94 °C (30 Re: revised manuscript submitted; one query from editor sec), 53 °C (30 s), and 68 °C (1 min); and then 72 °C (7 min). Amplified products were analyzed on 1% agarose gels.

### Transgenic events, elite varieties, and introgression lines

The parent variety, Williams 82, was developed by the USDA-ARS and the Illinois Agriculture Experiment Station and released in 1981. It is a late group III indeterminate variety (relative maturity 3.8). Williams 82 was selected because of its ability to facilitate plant genetic transformation. Williams 82 was also the cultivar used for soybean whole-genome shotgun sequence of *Glycine max*^73^. Three independent transgenic events of *psNTP9*, designated as 14A, 16C and 17B, were previously characterized^19^. Null siblings that did not contain *psNTP9* gene were segregated from T2 population of each transgenic event and used as negative control. In order to integrate *psNTP9* gene into newer varieties for commercial product development, lead events were backcrossed into several elite varieties differing in relative maturity. The soybean elite varieties chosen as recurrent parents for backcrossing, included A1900 (relative maturity 1.9), DSR262 (relative maturity 2.6), and A3431 (relative maturity 3.4).

### Multiple year and multiple location field trials

The field trials were conducted in the US from 2016 through 2019 as well as in Argentina in 2019. Except for 2017 seed increases and plot trials in Puerto Rico, field trials were located within the major soybean production areas of both Argentina and US, thereby covering the diverse environmental conditions for soybean in these two countries. All the trials used a Randomized Complete Block Design (RCBD). Due to seed limitation of transgenic events, 2016 trial sites used 3 replicates. The field trials in 2017 through 2019 had 4–5 replications, with plot size of 7.6 m × 3.0 m (or 25 ft × 10 ft). The four rows were sown at a row spacing of 0.76 m (or 2.5 ft); only the interior two rows of soybean plants were harvested for seed yield analysis. All the plant experiments were in compliance with relevant institutional, national, and international guidelines and legislation.

### Field trial data acquisitions

In the course of plant growth and development, agronomic performance data were collected for gene efficacy evaluation, in addition to soybean seed yield at harvest. Agronomic data included days to 50% emergence, early plant stand, initial plant density, seedling vigor scoring, plant height (at V2–V3 and R6–R7), days to 50% flowering, days to 50% maturity, lodging score, shattering score, plant stand at R8, flower color, seed moisture (%) and test weight at harvest, grain weight, arthropod counts, pest damage and disease infestation.

### Field trial statistical analyses

Agronomic and yield data from multiple locations over multiple years were analyzed using a linear mixed model. The locations were analyzed individually as well as grouped by country and a global analysis of all locations combined.

The individual location analysis used the following model:

$$y_{ij} = \, \mu \, + g_{i} + \, r_{j} + \, e_{ij} ,$$

$$r_{j} \sim \, iid \, N\left( {0,\sigma^{2} Rep} \right)\,{\text{and}}\,e_{ij} \sim \, iid \, N(0, \, \sigma^{2} plot).$$

- Notation:
  - y_ij_ denotes the unique individual observation.
  - *µ* denotes the overall mean.
  - *g*_i_ denotes the mean of the ith entry.
  - *r*_j_ denotes the effect of the jth block.
  - *Ε*_*ij*_ denotes the effect of the plot assigned the ith entry in the jth block (residual error).
  -  ~ iid *N*(0,* σ*^2^) indicates random variables that are identically and independently distributed (iid) as normal with zero mean and variance *σ*^2^.

The combined location analysis used the following model:

$$y_{ijk} = \, \mu \, + g_{i} + \, l_{j} + \, rk_{(j)} + \, \left( {gl} \right)_{ij} + \, e_{ijk} ,$$

$$l_{j} \sim \, iid \, N\left( {0, \, \sigma^{2} Loc} \right), \, rk_{(j)} \sim \, iid \, N\left( {0, \, \sigma^{2} Rep} \right), \, \left( {gl} \right)_{ij} \sim \, iid \, N\left( {0, \, \sigma^{2} Loc\mathop A\limits^{ \circ } \sim Ent} \right),\,{\text{and}}\,e_{ijk} \sim \, iid \, N(0, \, \sigma^{2} plot).$$

- Notation:
  - y_ijk_ denotes the unique individual observation.
  - *µ* denotes the overall mean.
  - *g*_*i*_ denotes the mean of the ith entry.
  - l_j_ denotes the effect of the jth location.
  - *rk*_(*j*)_ denotes the effect of the kth block within the jth location.
  - (*gl*)_*ij*_ denotes the interaction between the entries and locations.
  - *e*_*ijk*_ denotes the effect of the plot assigned the ith entry in the kth block of the jth location (residual error).
  -  ~ iid *N*(0,*σ*^2^) indicates random variables that are identically and independently distributed (iid) as normal with zero mean and variance *σ*^2^.

The mixed model analyses of variance were conducted using SAS Proprietary Software version 9.4 (SAS Institute, 2015). The level of statistical significance was predetermined to be 5% (*p* = 0.05).

### Transcriptome analyses of field-grown and greenhouse-grown WT and transgenic soybean lines

For RT and qRT-PCR analyses of mature leaf tissue from field-grown soybean lines (including Williams 82 WT, and the three transgenic lines, 14A, 16C and 17B), the harvested leaves were frozen, and leaf discs (0.5 cm in diameter) were excised from them, frozen and shipped frozen to the University of Texas. For each analysis, three leaf discs were sampled and ground frozen by mortar and pestle. Texas Crop Science, LLC contracted the field trials and had permission to collect the soybean leaves.

For RT-PCR and qRT-PCR, 1 μg total RNA was extracted using the Spectrum Plant Total RNA kit (Sigma) for semi-RT PCR. The RNA was converted to cDNAs by using the High Capacity cDNA Reverse Transcription kit (Applied Biosystems) with 10 × RT Random Primers (Applied Biosystems). For RT-PCR, reaction mixtures contained 25 μL of Quick-Load Taq 2 × Master Mix, 1 μL of 10 µM forward primer, 1 μL of 10 µM reverse primer and 2 μL of cDNA in final volume of 50 μL. The PCR protocol had an initial denaturation at 95 °C for 30 s, and 36 cycles of denaturation at 95 °C for 30 s, annealing at 53 °C for 1 min, and extension at 68 °C for 1 min (*BAR*, *TUB*) or 1.5 min (*psNTP9*). The final extension was at 68 °C for 5 min. After PCR, 1% agarose gel was used for electrophoresis. RT-PCR primers used are found in Supplementary Table S1 of Veerappa et al.^19^.

For RNA-seq and qRT-PCR analyses of DE genes of greenhouse-grown WT and transgenic plants, total RNA was extracted from frozen leaf tissues of 45-day-old (V5 developmental stage) 14A and WT plants grown in the greenhouse without supplemental lighting (14 h light). Care was taken to make sure that all the leaves harvested were the same size and developmental stage and that the plants were also healthy and at equivalent developmental stages and age. Total RNA was extracted from frozen tissues using Sigma Purelink RNA isolation kit. An Invitrogen DNaseI kit was used for digestion of genomic DNA, and a High capacity cDNA synthesis kit (AB Biosystems) was used for cDNA preparation.

qRT-PCR primers for *psNTP9* are found in Supplementary Table S1. qRT-pCR primers for the reference gene *ELF1B* are found in Supplementary Table S2. qRT-PCR primers for the DE target genes are found in Supplementary Table S2**.** Primers were designed using NCBI Primer-BLAST^75^ so that one primer of each pair spanned an exon-exon junction, preventing amplification of gDNA. Reactions (20 µL) contained 5 μL of cDNA (1 ng/µL), 0.4 µL of each primer (10 μM), 10 μL of Power SYBR Green master mix (Applied Biosystems, USA), and 4.2 μL nuclease-free water. qRT-PCR was conducted using a ViiA7 Real-Time PCR System (Thermofisher Scientific) as follows: 95 °C for 10 min, followed by 40 cycles of 95 °C for 30 s, 58 °C for 30 s, 72 °C for 30 s in 96-well optical reaction plates (Applied Biosystems, USA). Expression of reference gene *ELF1B*^74^ was used to normalize target gene expression in three replicates. Relative expression was calculated using the DDCT method^76^. Dissociation curve analyses were used to check for amplification of homogenous products.

For RNA-seq analyses, cDNA library preparation and sequencing (Illumina HiSeq 2500; paired-end, 75 bp reads) was carried out by the Genome Sequencing and Analysis Facility (GSAF) at the University of Texas at Austin. Post-sequencing workflow was as described by Veerappa et al.^19^. An average of 282 million reads were mapped for each sample, with 98.2% of reads mapping to exons + introns in the reference genome (assembly: Gmax_508_v4.0; annotation: Gmax_508_Wm82.a4.v1^77^). Genome files were retrieved from the JGI Genome Portal (https://data.jgi.doe.gov/refine-download/phytozome?organism=Gmax&expanded=508). Differentially-expressed genes (DEG), defined as having fold-change (FC) expression ≥ 1.5, relative to WT expression values, were identified using the R Bioconductor module DESeq2 (*padj* ≤ 0.05^78^).

### GO term enrichment analyses

Analyses of up- or down-regulated DEG lists for GO BioProcess term enrichment were conducted using the Soybase GO Term Enrichment Tool (https://soybase.org/goslimgraphic_v2/dashboard.php). Overrepresented processes were defined as being enriched ≥ fivefold (*p* ≤ 0.05, Bonferroni correction). *Arabidopsis thaliana* Col database resources within BioCyc (https://biocyc.org/ARA/) were used to retrieve genes, annotation and pathways information related to cuticle development, an overrepresented GO BioProcess. Soybase genome annotation (Wm82.a4.v1) data were used to identify soybean orthologs of Arabidopsis genes.

### Western blot analysis of psNTP9 protein extracted from different tissues of 3 soybean lines

Tissue was harvested from either greenhouse-grown or field grown WT, 14A, 16C, and 17B soybeans, and frozen. Frozen tissues were homogenized with a bead beater or with mortar and pestle and immediately dissolved in 150 μL of protein isolation buffer containing 10 mM Tris–HCl pH 8.0, 1 mM EDTA, 0.5 mM EGTA, 1% Triton, 0.1% SDS, 140 mM NaCl, 5 mM β-mercaptoethanol. Protein quantity was measured with Bradford assay, using the BioRad reagent, and equal loading was assayed by actin immunostaining. Proteins were separated by SDS PAGE and transferred to a PVDF membrane by semi-Dry transfer method. Blots were incubated with 8B6 monoclonal antibody (GeneScript; 1:50 dilution) and goat anti-mouse fluorescent antibody (1:5000 dilution). Fluorescent images were detected using Odyssey infrared imaging system (Licor). Although the full length of each blot was imaged, only the portion of each blot above the 25 kDa marker is presented in the Results, since no immunostaining was detected below this portion in any of the transgenic lines.

### Total chlorophyll measurement

Fresh leaves of thirty-four-day-old greenhouse grown wild type and transgenic plants at V3 stage (the third trifoliate stage) were ground using liquid nitrogen. The photosynthetic pigments were extracted using 80% acetone. Total chlorophyll content was determined using a spectrophotometer following the protocol of Fritschi and Ray^79^.

### Determination of soluble leaf proteins

Fresh leaves of thirty-four-day-old greenhouse grown wild type and transgenic plants at V3 stage (the third trifoliate stage) wild type and transgenic plants were frozen in liquid nitrogen. The soluble leaf protein content was determined by the method of Bradford^80^.

### Cuticular transpiration and chlorophyll leaching assays

V5 stage leaves of well-watered Williams 82 (wild type) and independent *psNTP9* transgenic lines (14A and 17B) were used for cuticular transpiration and chlorophyll leaching assays. For the cuticular transpiration assay, plants were placed in the dark for 6 h to facilitate stomatal closing, then leaves were detached and 0 h weights were recorded. Three replicates were placed in darkness at room temperature and 40% relative humidity. Leaves were then weighed at 1 h intervals for 7 h. Leaf weights at each time point were calculated as percentages of 0 h leaf weights.

For the chlorophyll leaching assay, V5 stage soybean leaves of well-watered plants were detached and immersed in 40 mL of 80% (v/v) ethanol in a 50 mL capped tube, then placed on a rocker platform in the dark. A series of 100 μL aliquots were taken after 3, 4, and 4.5 h immersion and extracted total chlorophyll was calculated as µmol chlorophyl = [7.93 × A_664_ + 19.53 × A_647_], according to Lee et al.^48^. Chlorophyll leaching at each time point was calculated as percentages of the total chlorophyll extracted at 24 h after immersion.

### Purification of cell wall proteins and of nuclei from etiolated soybean seedlings

Wall proteins were purified from hypocotyl tissues of etiolated 6-d-old soybean seedlings, using the method of Kim et al.^51^. As a negative control to test for cytoplasmic contaminants in the wall extract, the preparation was tested by immunoblot analysis for the presence of actin. Nuclei were purified by the method of Chen et al.^24^, using hook tissue from 6-day-old etiolated soybean seedlings.

### Microscopic analyses of leaves

For determination of determination of cuticle and wall thickness transmission electron microscopy (TEM) was used. Leaves (V5 developmental stage) of approximately the same size (8–10 cm length) were cut from 45-day-old 17B and WT plants grown in the greenhouse. Small pieces approximately 1 × 1 cm were excised from each leaf and immersed overnight at room temperature in a mixture of EM-grade aldehydes containing 4% glutaraldehyde and 2% formaldehyde in 0.05 M cacodylate buffer, pH 7.4. After three buffer rinses, the pieces were cut into 1 mm strips which were then immersed overnight at room temperature in 0.5% Ruthenium Red in 0.1 M cacodylate buffer. The strips were then washed with cacodylate buffer and immersed in reduced osmium (2% osmium tetroxide and 2% potassium ferrocyanide in 0.1 M cacodylate buffer, pH 7.4). Osmium fixation of the strips was started in a preparative microwave and was completed with 2 h immersion on ice. Following dehydration in an ethanol series, the leaf pieces were embedded in Epon Hard 812 (Electron Microscopy Sciences, https://www.emsdiasum.com), from which 70 nm thin sections were cut with a diamond knife, picked up on Formvar-coated grids, and imaged with a Tecnai T12 TEM operated at 80 kV. During thin sectioning, care was taken to position the diamond knife perpendicular to the leaf surfaces to insure accuracy of subsequent measurements.

Measurements of cell wall and cuticle thickness were made from TEM images at instrumental magnification of 26,500× and 43,000× with Image J, using the scale embedded in each image to calibrate the line tool. Measurements were made approximately every 500 nm, typically three to five measurements were taken at equal intervals across each image for both the cuticle and cell wall. For each cell measured, the thickness of the cuticle and wall was measured by lines originating at and perpendicular to the cuticle-cell wall junction. It was observed that the wall thins near cross walls, so measurements were not taken from these regions.

For determination of stomatal density and stomatal coverage, leaf peels (20–25 per line) were taken from the abaxial surface of soybean leaves (V5 developmental stage) 14A, 17B and WT plants grown in the greenhouse. Peels were immediately transferred to glass slides with 100 µL of Arabidopsis leaf buffer (10 mM KCL, 25 mM MES, pH 6.15). The prepared slide was then imaged with a 40 × objective lens. Image analysis was conducted using ImageJ. First, the area in each peel with the stomatal layer both visible and in focus was calculated and then a measurement of the area of each stoma was taken with the polygon tool. The total area of stomata visible over total peel area determined the percent area of stomatal coverage and the number of stomata present over the total visible area of a particular peel determined the stomatal density.

For trichome length analyses, similar-sized leaves (8–10 cm) from the growing tips of wild-type and 17B plants were collected. The leaves were cut into 1 cm strips, perpendicular to the midrib, and the strips were stored in 70% ethanol overnight. The cleared leaf pieces were placed in 50% bleach until they were colorless and then transferred to water.

Leaf pieces were then held between two glass slides with large spring clips which flattened and collapsed the trichomes into a single image plane. Using light microscopy, pictures of the flattened trichome samples were captured. Measurements of trichome length were carried out using ImageJ. As many trichomes were measured as possible to get a representative distribution. Approximately 200 trichomes of each group were measured (total n = 817), representing about 70% of the total trichomes visible. Certain trichomes were difficult to follow from start to end through the entirety of the trichome and thus were not included. Statistical analysis was carried out using the student’s *t*-test.

## Supplementary Information

Supplementary Information 1.

Supplementary Information 2.

## Data Availability

RNA-seq data were deposited in the NCBI Sequence Read Archive (SRA) under accession number PRJNA765 https://www.ncbi.nlm.nih.gov/sra/?term=PRJNA765378.
