# Supplementary material for: Constitutive expression of a pea apyrase, psNTP9, increases seed yield in field-grown soybean
Source: Sci Rep. 2022 Jun 27;12:10870. doi: 10.1038/s41598-022-14821-7 (PMC9237067; doi:10.1038/s41598-022-14821-7)
Supplement: Supplementary file 1 — Supplementary Information 1. [file 41598_2022_14821_MOESM1_ESM.pdf]

**Fig. 5 b**

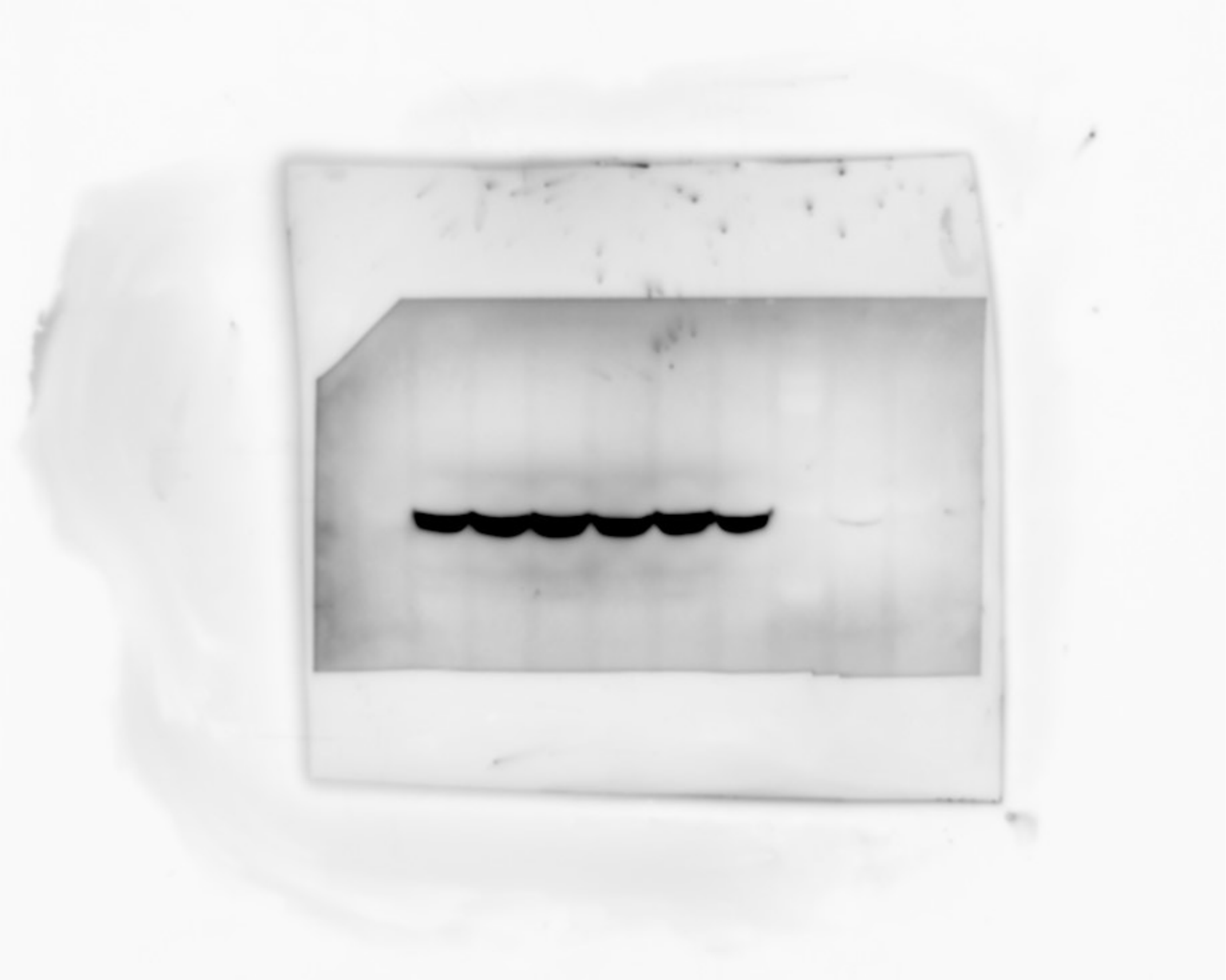

**Fig. 5 b**

*Molecular mass standards*

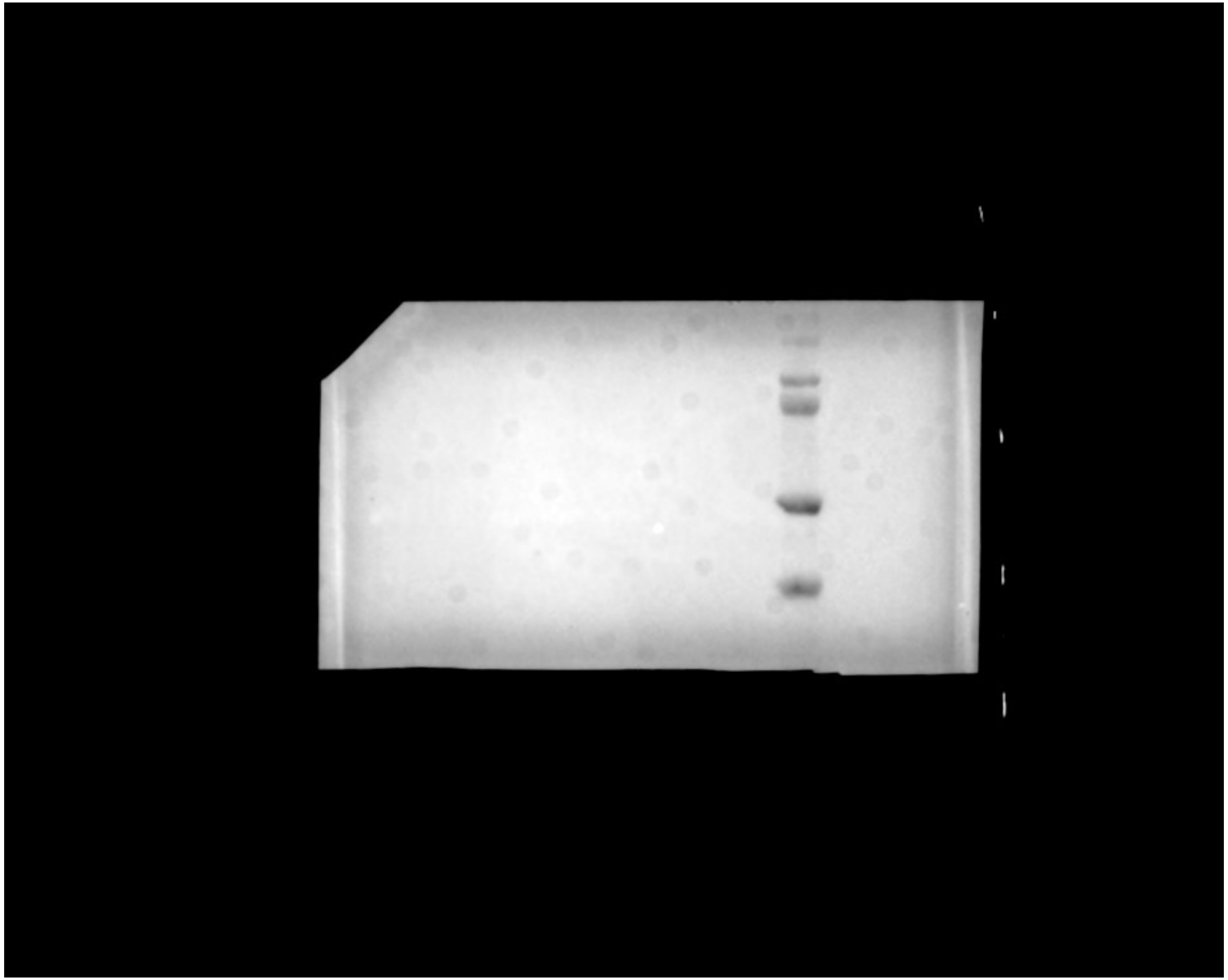

**Fig 5. c**

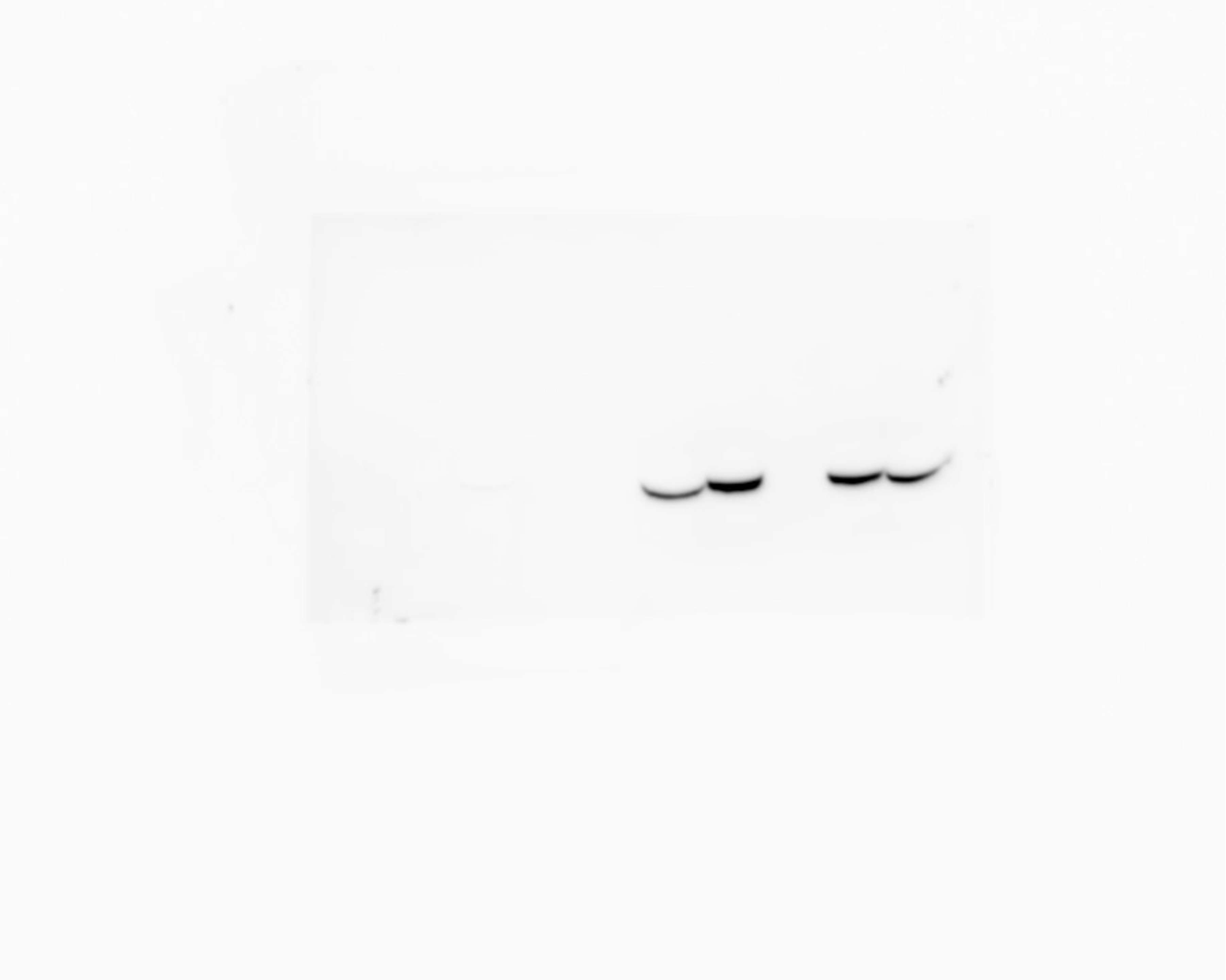

# Fig 5. c

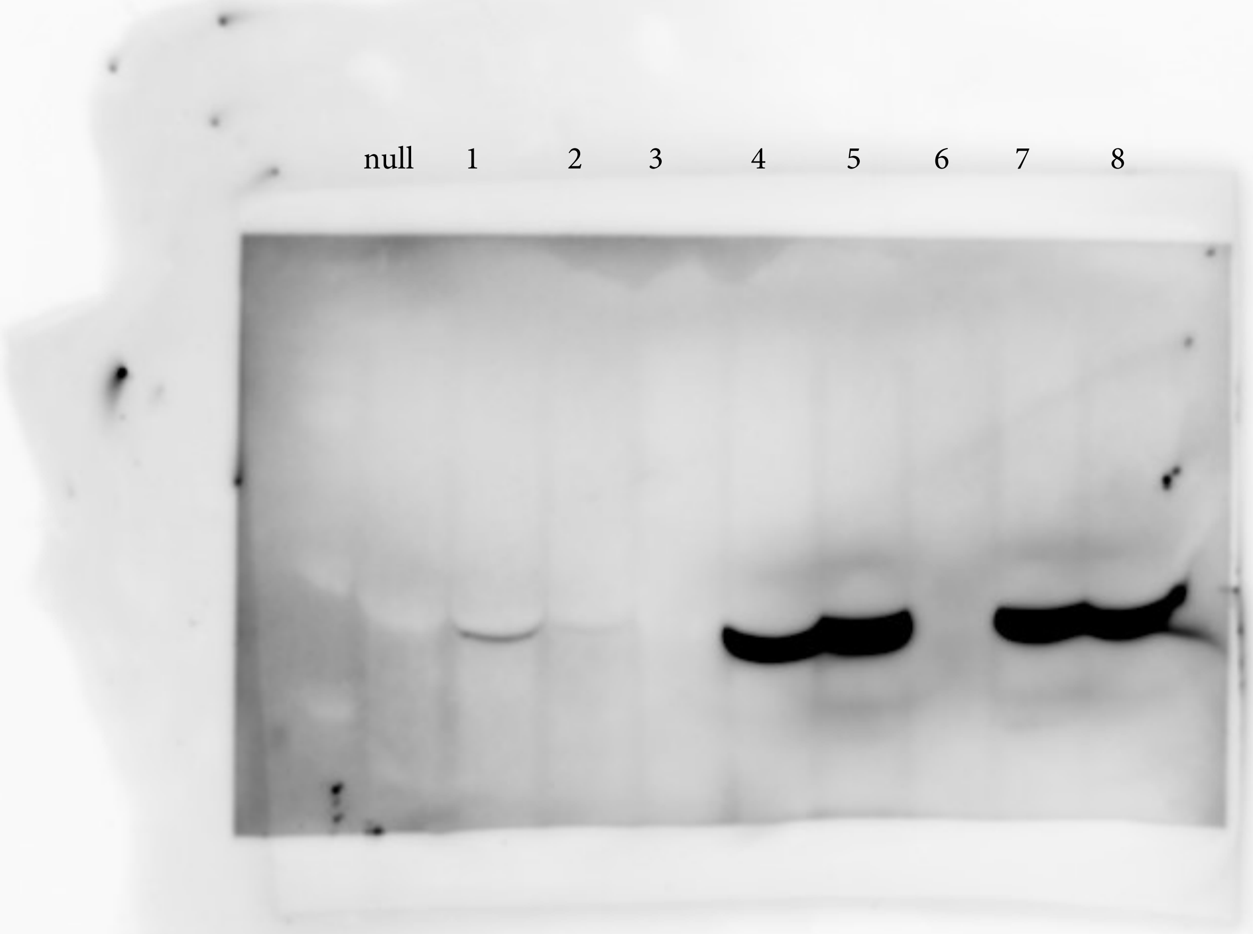


This is a higher sensitivity version of the blot shown in image 3 and in Fig. 5c of manuscript, where lane identities are given.

**Fig 5. c**

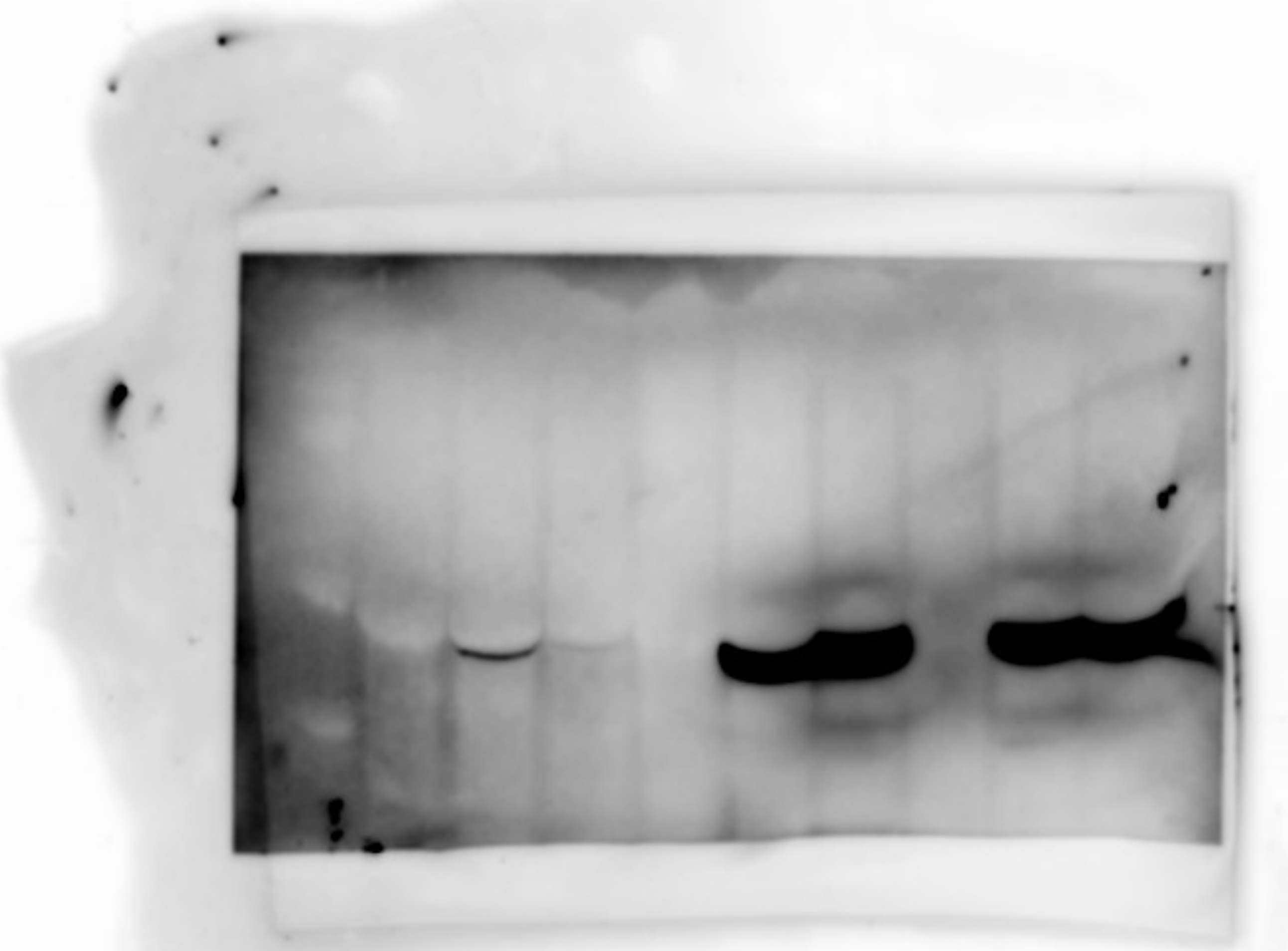

Fig 5. c

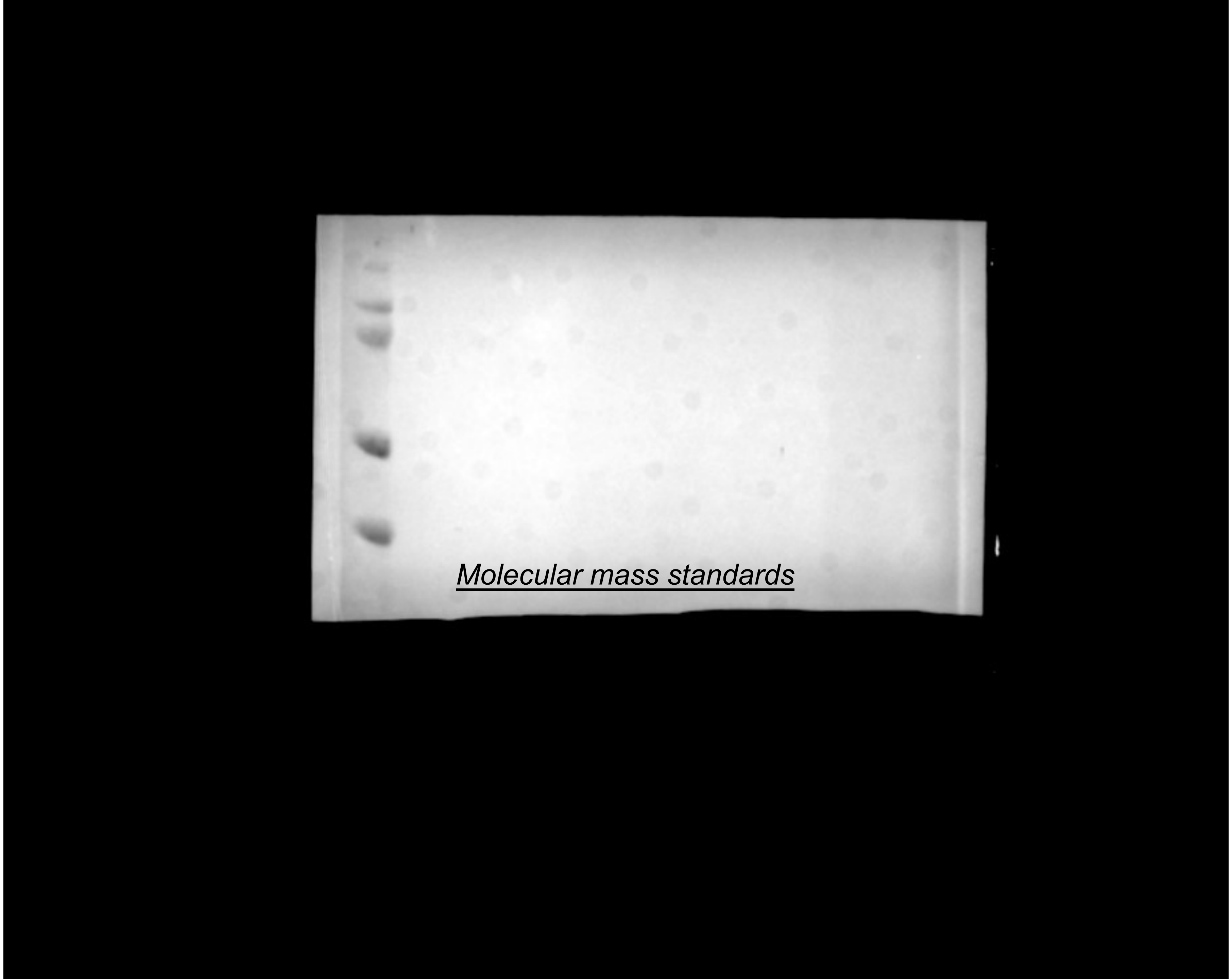

**Figure S1B**

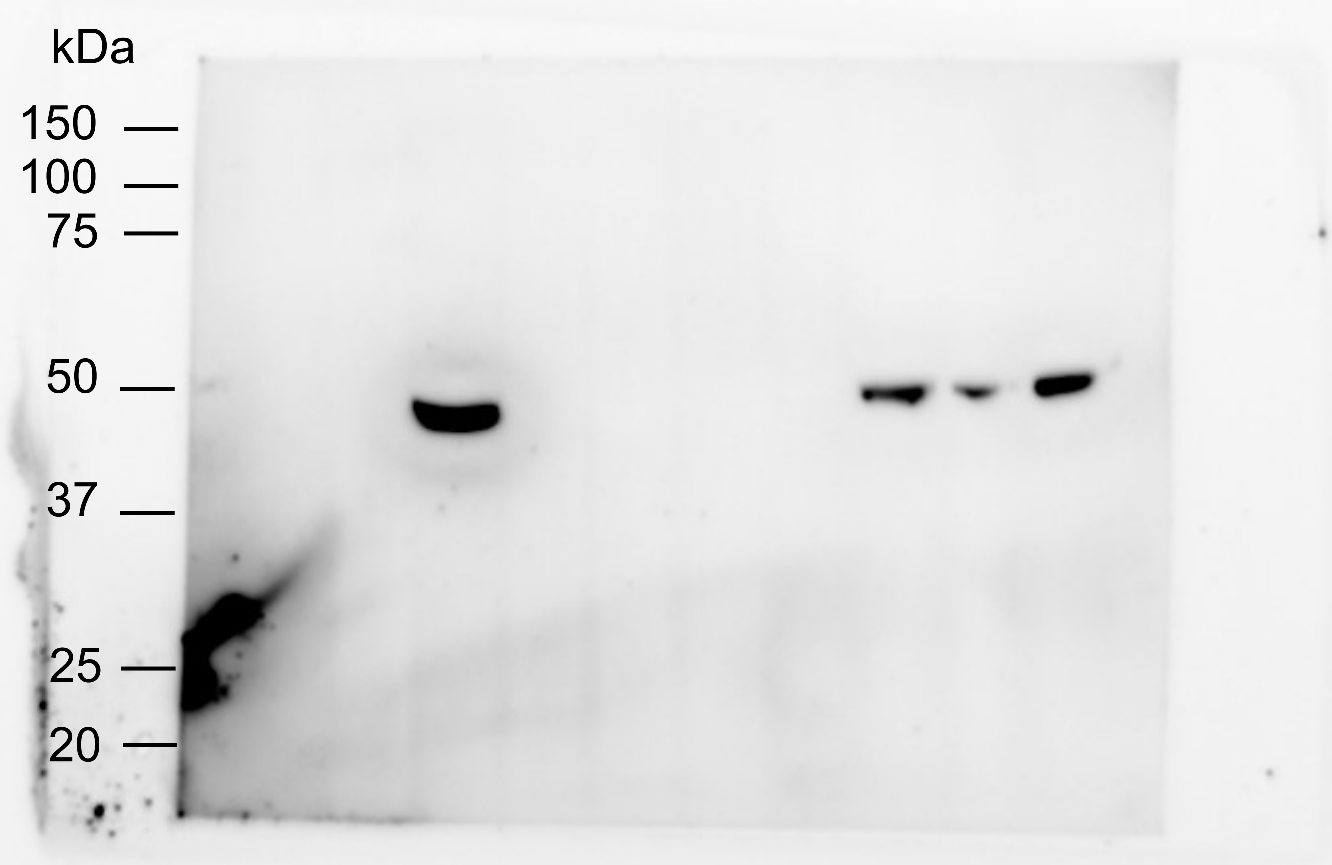

# Figure S1B

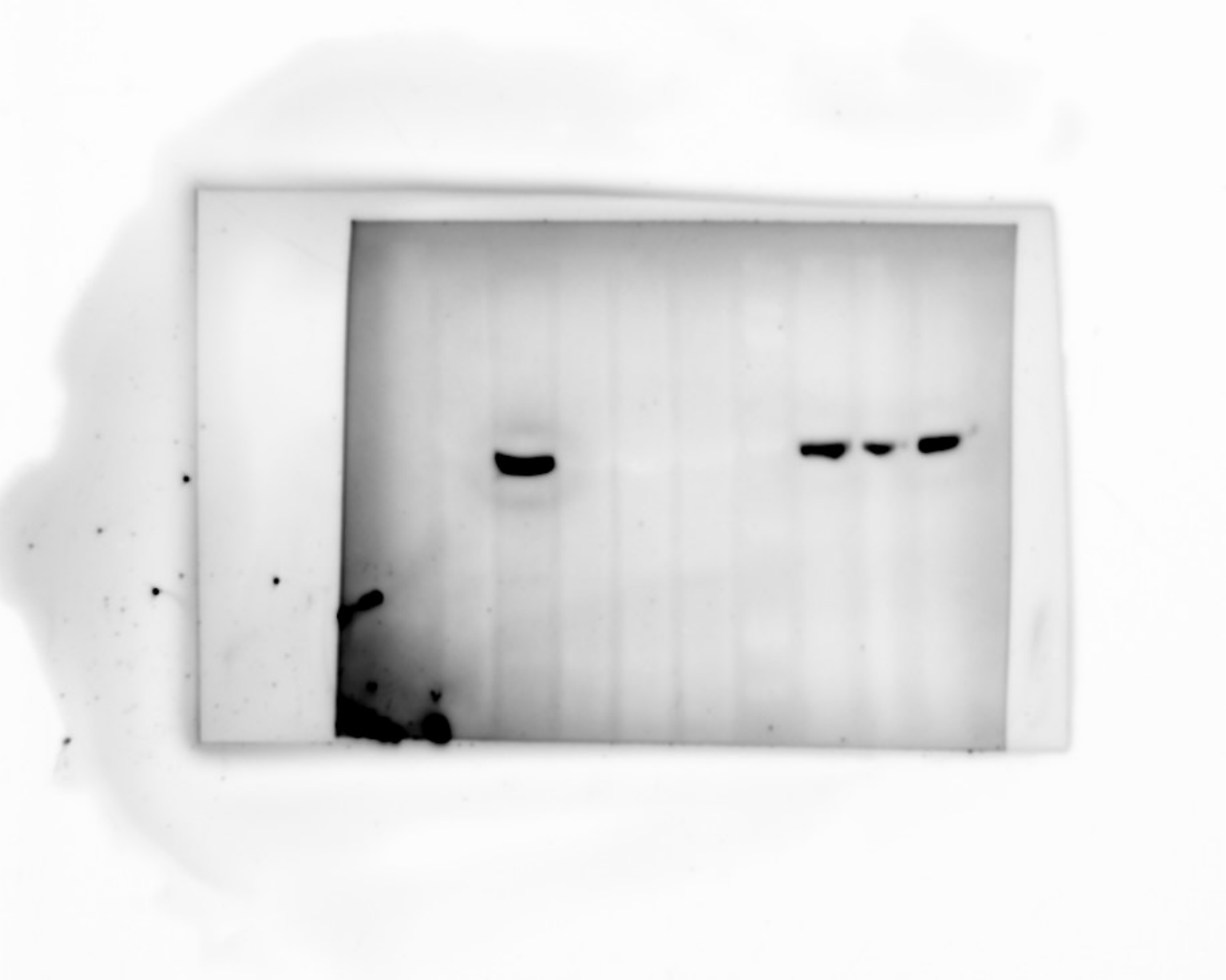

# Figure S1B

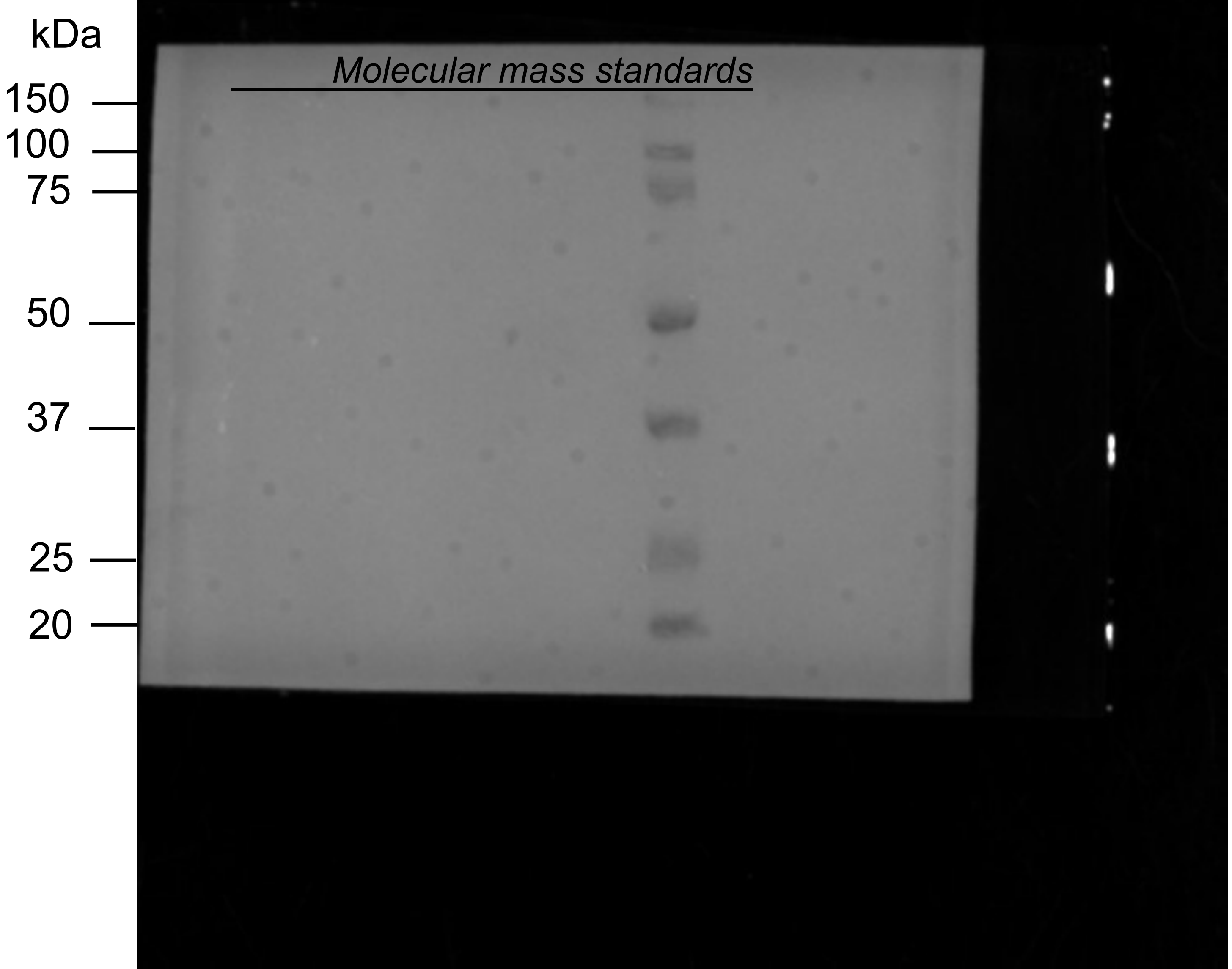

# Figure S2

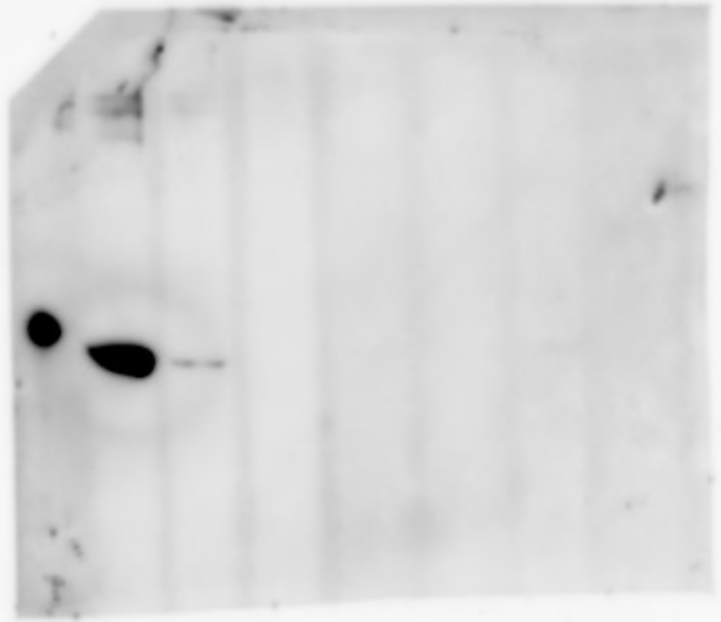

*Note: lane on far left had 2 ng of purified psNTP9 and was removed from Fig. S2.*

# Figure S2

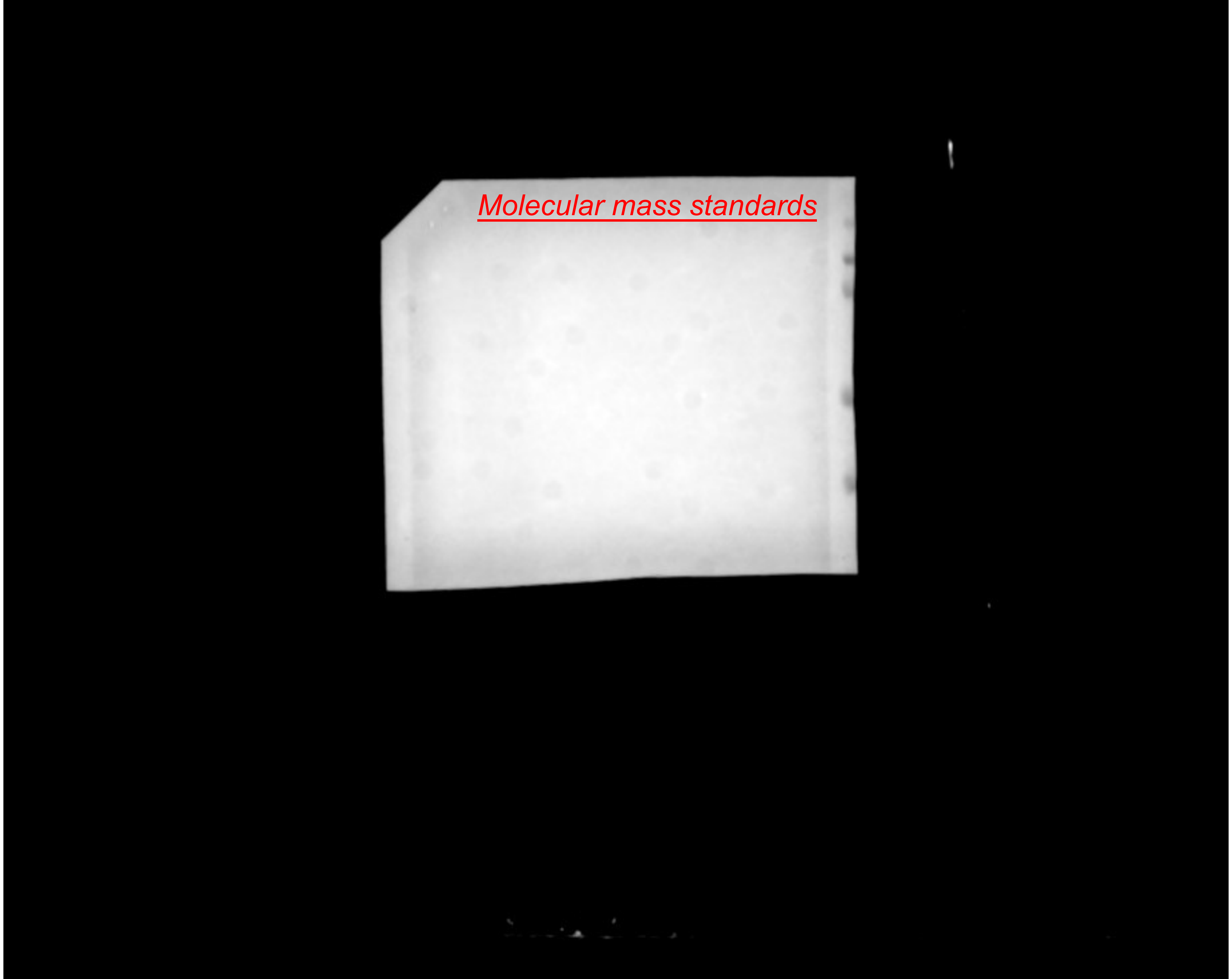

# Suppl. Figure S3A with image full-length Coomassie-blue stained SDS-PAGE from which the proteins in the blot were transferred

*Note only this part of the gel was transferred for Western blot analysis

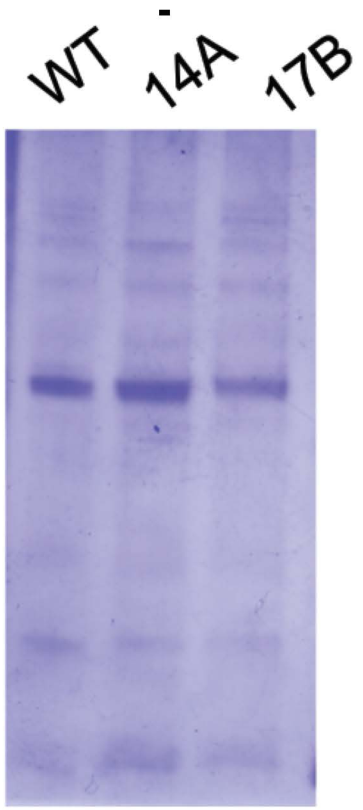


Coomassie-stained SDS-PAGE after transfer

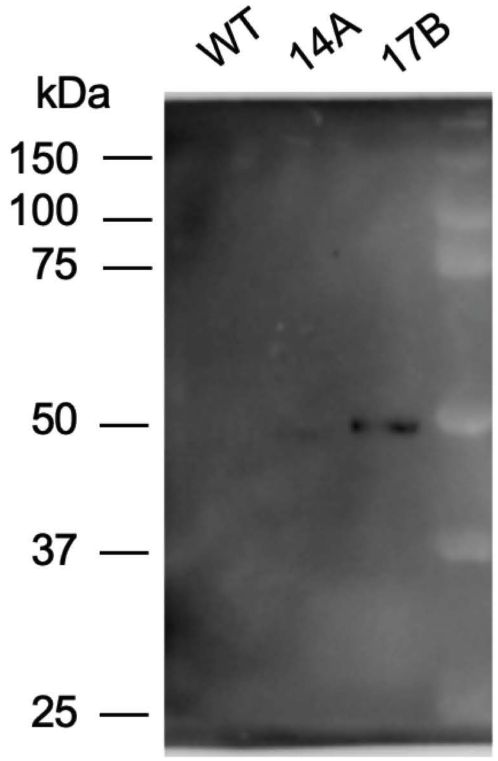


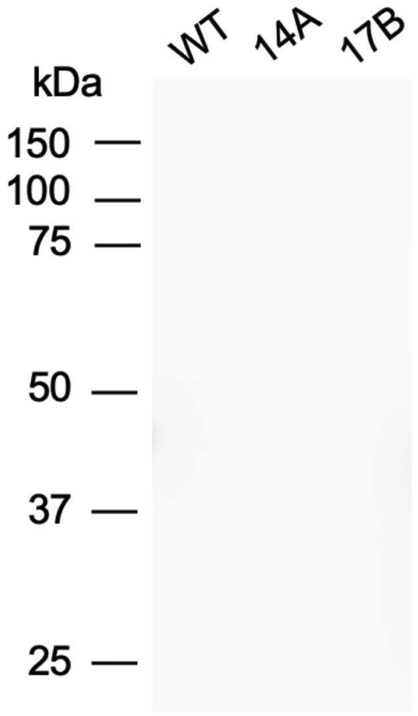


Blot was stripped and re-probed with anti-actin

# Figure S3A

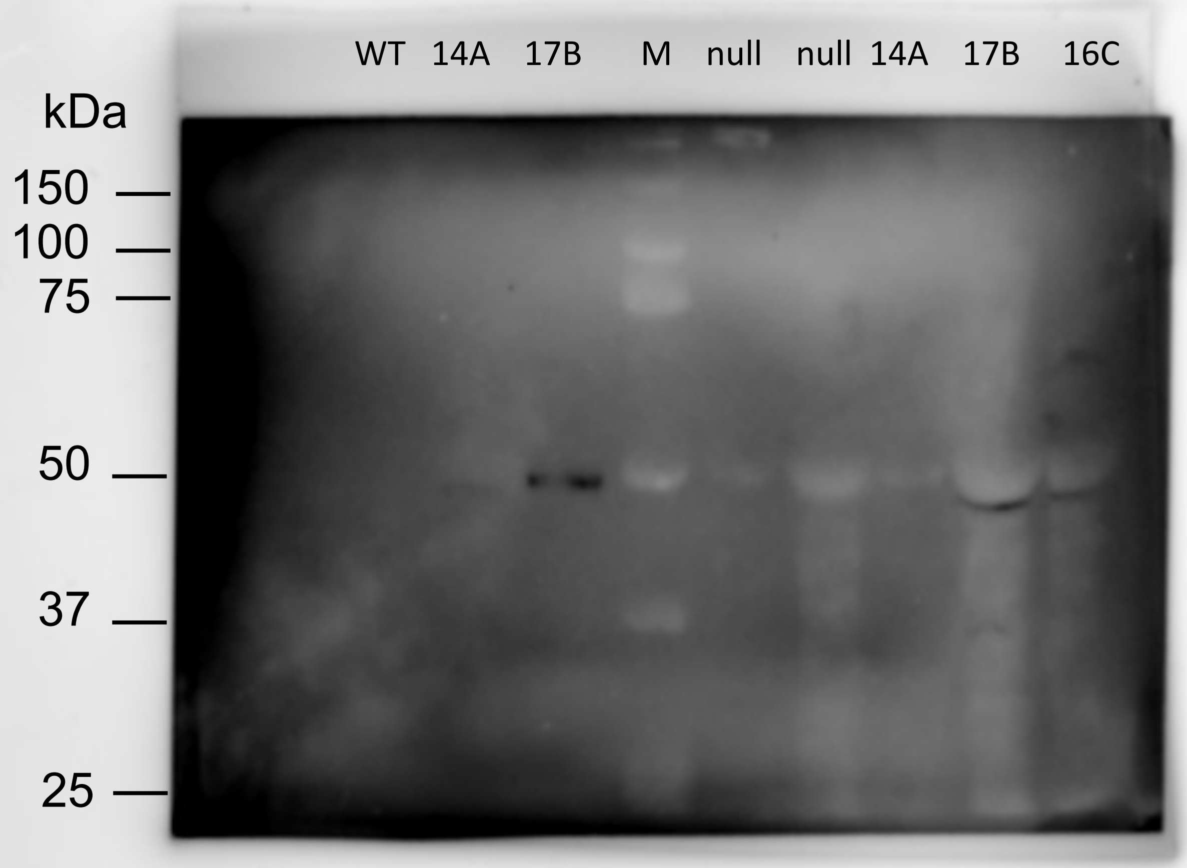


Note: This is the original blot for Figure S3A, but for that Figure, only the WT, 14A, 17B and marker (M) Lanes on the left side were presented.

# Original full-length blot of Suppl. Figure S3B--purified nuclei

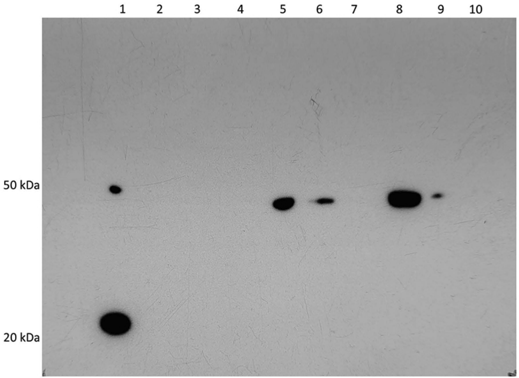


Western Blot:
Lane 1: Pure apyrase (5 ng)
Lane 2: Marker
Lane 3: Wildtype Hook Nuclei
Lane 4: Blank
Lane 5: 14A
Lane 6: 14A 1/10
Lane 7: 14A 1/100
Lane 7: Blank
Lane 8: 17B Hook Nuclei
Lane 9: 17B 1/10
Lane 10: 17B 1/100

Mass spectroscopy analysis showed that the 25 kDa protein immunostained in lane 1 was a proteolytic breakdown produce of psNTP9 (see below)

## Mass spectroscopy of the protein in the 25 kDa band detected these psNTP9 peptides:

MELLIKLITF LLFSMPAITS SQYLGNNLLT SRKIFLKQEE ISSYAVVFDA GSTGSR**IHVY** **HFNQNLDLLH**
**IGKGVEYYNK** **ITPGLSSYAN** **NPEQAAKSLI** **PLLEQAEDVV** **PDDLQPK**TPV RLGATAGLRL LNGDASEKIL
QSVRDMLSNR STFNVQPDAV SIIDGTQEGS YLWVTVNYAL GNLGKKYTKT VGVIDLGGGS VQMAYAVSKK
TAKNAPKVAD GDDPYIKKVV LKGIPYDLYV HSYLHFGREA SRAEILKLTP RSPNPCLLAG FNGIYTYSGE
EFK**ATAYTSG** **ANFNK**CKNTI RKALKLNYPC PYQNCTFGGI WNGGGGNGQK NLFASSSFFY LPEDTGMVDA
STPNFILRPV DIETKAKEAC ALNFEDAKST YPFLDKKNVA SYVCMDLIYQ YVLLVDGFGL DPLQKITSGK
EIEYQDAIVE AAWPLGNAVE AISALPKFER LMYFV

# Detection of psNTP9 in soybean leaf extracts

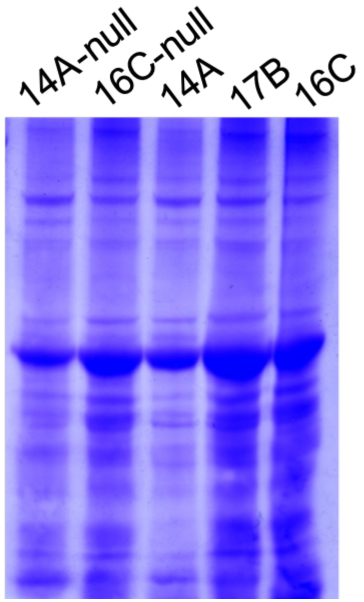


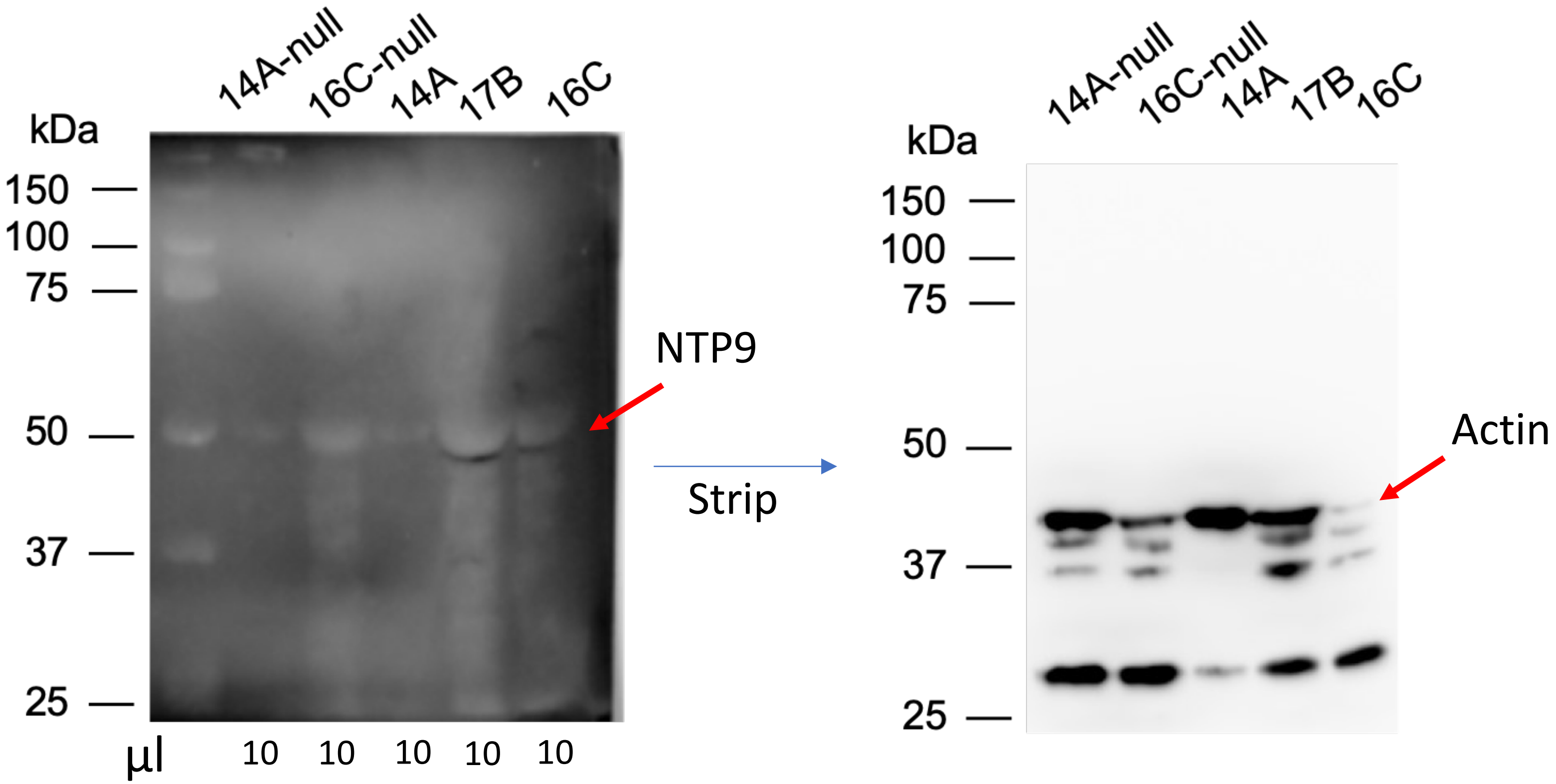


8B6 1:50/ Goat anti-mouse-HRP

Anti-actin/ Goat anti-mouse-HRP

The WB were using another fresh prepared soybean leaf extracts for detecting NTP9 signal in 14A.
I might have to harvest new batch of leaves of 14A for the future experiment.
