# Supplementary material for: Constitutive expression of a pea apyrase, psNTP9, increases seed yield in field-grown soybean
Source: Sci Rep. 2022 Jun 27;12:10870. doi: 10.1038/s41598-022-14821-7 (PMC9237067; doi:10.1038/s41598-022-14821-7)
Supplement: Supplementary file 2 — Supplementary Information 2. [file 41598_2022_14821_MOESM2_ESM.pdf]

Supplementary Figures and Tables

Constitutive Expression of a Pea Apyrase, *psNTP9*, Increases Seed Yield in Field-Grown Soybean

Tanya Sabharwal[1], Zhongjin Lu[2], Robert D. Slocum[3], Seongjoon Kang[1], Huan Wang[1], Han-Wei Jiang[1], Roopadarshini Veerappa[1], Dwight Romanovicz[1], Ji Chul Nam[1], Simon Birk[1], Greg Clark[1], Stanley J. Roux[1, 4]

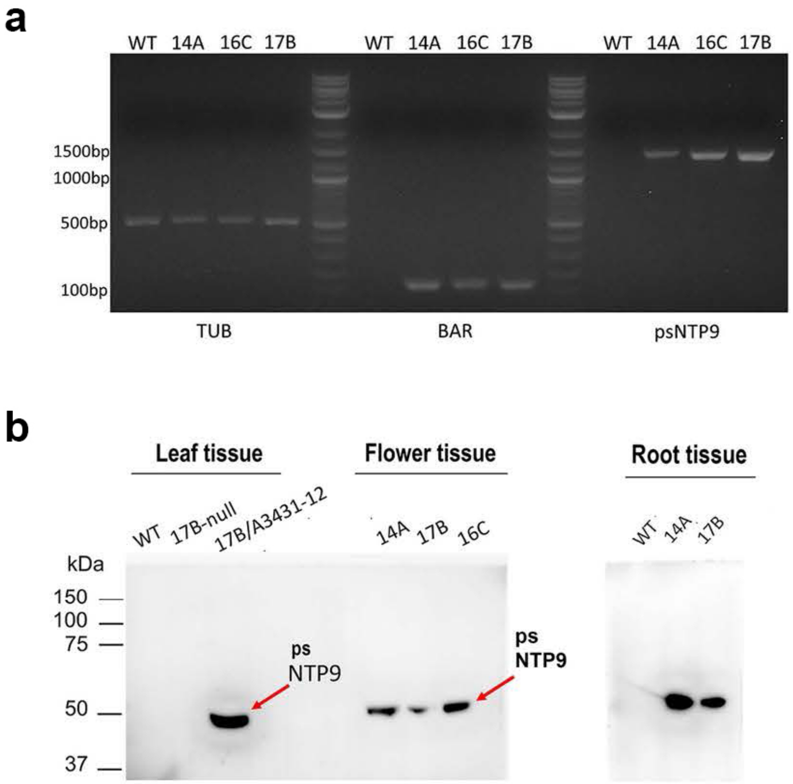

Supplementary Figure S1. a) RT-PCR of RNA isolated from leaves (V5 developmental stage) of field-grown 14A, 16C, and 17B lines of soybeans indicates that *psNTP9* transcripts were being expressed in all three lines. *Tubulin (TUB)* and *BAR* gene expression was used to confirm equal loading . b) Immunoblots of leaf, flower and root tissues harvested from greenhouse-grown soybean plants at the V5 developmental stage reveal that the psNTP9 protein is immunostained in all three tissues by the 8B6 mAb. The blot also shows that *psNTP9* is expressed in the flower and root tissues of the 14A and 17B transgenic lines and in the flower tissue of the 16C transgenic line.

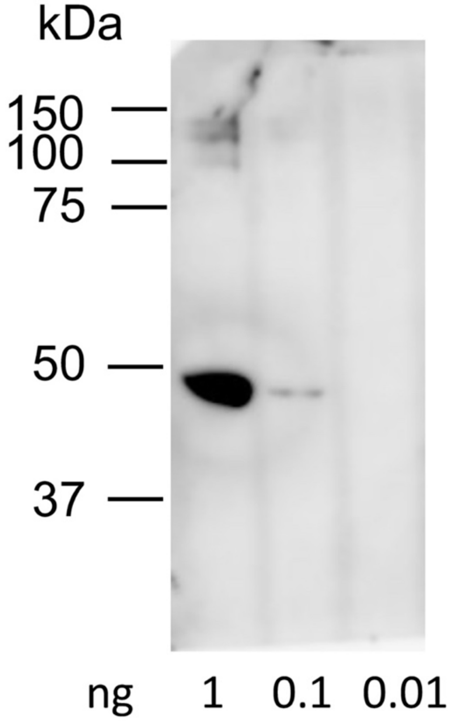

Supplementary Figure S2. The minimal level of purified psNTP9 detectable in immunoblots using mAb 8B6 as the primary antibody is approximately 0.1 ng. Each of the three psNTP9 samples assayed was mixed with 21 µg crude leaf protein extracts of wild-type soybean seedlings immediately before the samples were prepared for SDS-PAGE electrophoresis.

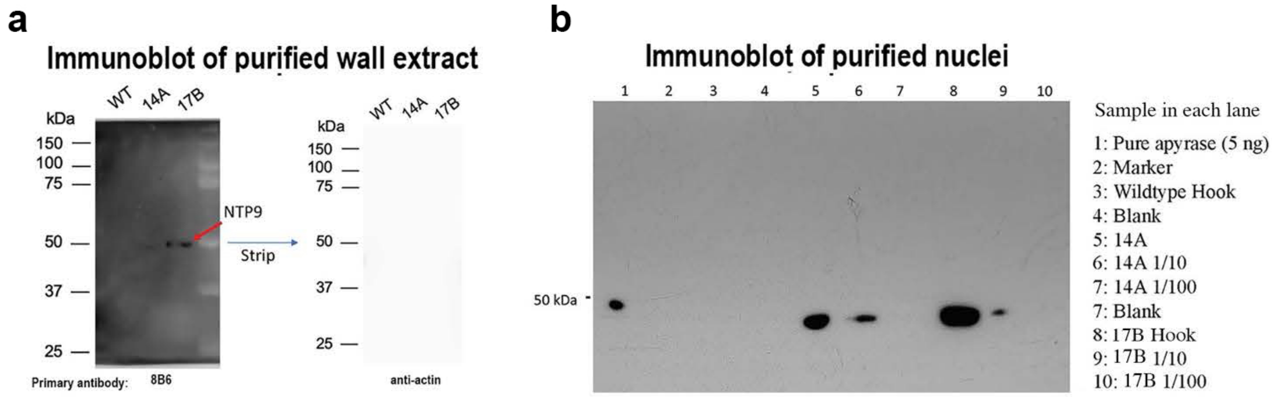

Supplementary Figure S3. Immunoblots of cell wall protein preparations from hypocotyls (a), and of nuclei isolated from the apical hook region (b), both from etiolated 6-d-old seedlings of 14A and 17B soybeans, using 8B6 antibodies. The only immunostaining band in both preparations is the 48 kDa psNTP9. In panel a, the blot was stripped and re-probed with anti-actin antibodies to test for cytoplasmic contaminants in the preparation. In panel b, purified pea apyrase was used as a positive control in lane 1.

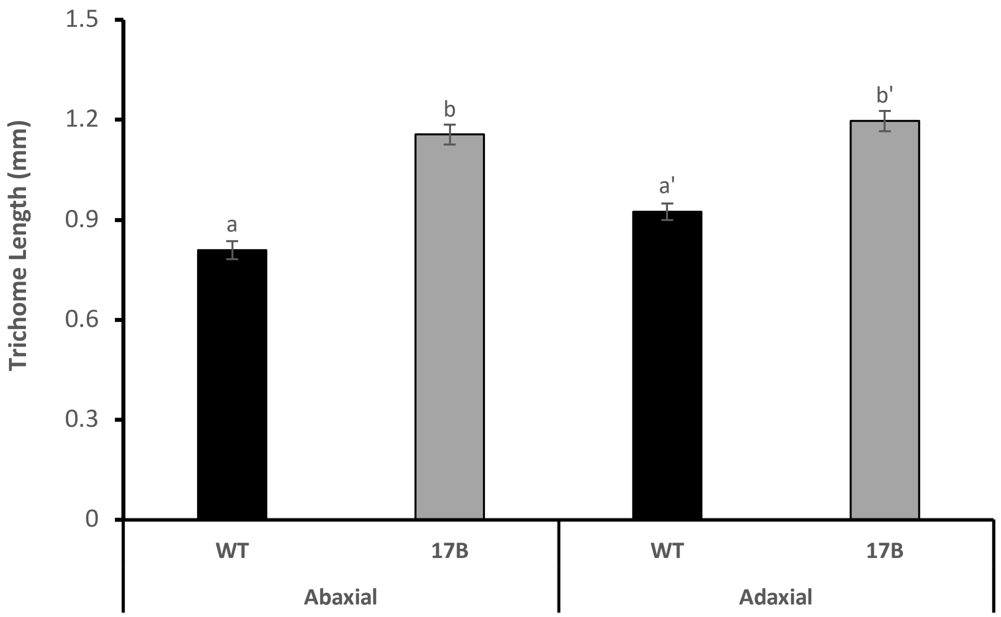

Supplementary Figure S4. Average trichome lengths on abaxial and adaxial surfaces of the wild-type and transgenic 17B leaves. Trichomes were longer on both abaxial and adaxial surfaces of 17B leaves compared to wild-type leaves. Values represent means ± S.E. Different letters above the bars indicate values that are significantly different from one another ($p < 0.05$).

Supplementary Table S1. PCR primers used for mapping of T-DNA insertion sites.

PCR experiment	Name	Sequence (5'-3')
**Probe labeling**	BarF	GCGTTCAAAAGTCGCCTAAG
	BarR	GCAAGACCGGCAACAGGATT
**TAIL-PCR**	LB1	TCACCGAAATCTGATGACCCCTAGAGTCAAGC
	LB2	TTCTTAAGATTGAATCCTGTTGCCGGTCTTGC
	LB3	GATGGGTTTTTATGATTAGAGTCCCGCAATTATAC
	AD1	NGTCGASWGANAWGAA
	AD2	TGWGNAGSANCASAGA
	AD3	AGWGNAGWANCAWAGG
	AD4	STTGNTASTNCTNTGC
	AD5	NTCGASTWTSGWGTT
	AD6	WGTGNAGWANCANAGA
**Inverse PCR**	LB1	TCACCGAAATCTGATGACCCCTAGAGTCAAGC
	Bar3R	CCAGAAACCCACGTCATGCC
	NTP1R	GTAGTATTCGATGCTGGTAGCAC
	NTP5F	TAATTGATGGAACCCAAGAAGG
	LB2	TTCTTAAGATTGAATCCTGTTGCCGGTCTTGC
	Bar2R	AGCCAGGGATAGCGCTC
	NTP6F	GGAGGAGGAAATGGACAGAAA
	35SproR	GTCCTCTCCAAATGAAATGAAC
**Validation PCR**	35SproR	GTCCTCTCCAAATGAAATGAAC
	Gm11R	CGTTGTTAAATAAGACAGTGACCCACA
	Gm03F	CATAGCAATCGTCTCACCATCCCCA
	Gm14R	ACGCAGTGGAACTTCTCTGATCAAAC
	LB3	GATGGGTTTTTATGATTAGAGTCCCGCAATTATAC

Supplementary Table S2. qRT-PCR verification of RNA-seq expression values for selected DE genes in 14A and 17B lines. PCR primers used for each gene are indicated.

Gene ID	Symbol	Annotation	Fold-Change Expression			Primers (5'-->3')	
			RNA-seq (14A)	qRT-PCR			
				14A	17B	Forward	Reverse
Cuticle Formation							
Glyma.17G069100	CER3	Fatty acid hydroxylase superfamily; ECERIFERUM 3	1.7	4.4	25.3	TGTACCAGAGTCTTTTACTGCTGGA	GAACAATTTCAACATTGCTGTGGC
Protein Synthesis							
Glyma.15G075600	RPS6B	40S ribosomal protein S6-2	1.5	3.6	45.8	CGCCTGTAATTCAAAAACCCTAA	CCCAAAATGCTCGTAGCTTCA
Stomatal Development							
Glyma.13G040100	SPCH	Transcription factor SPEECHLESS	-2.0	-1.4	-1.2	TTCGTTCCACATCGCCAGAG	GATGCTTGGTCTCCCCGTTT
Other							
Glyma.13G088700	ANN1	Annexin 1	1.5	2.4	3.2	CCGAAAAGCCTTCTCAGGTTGG	GATAAGCTTCCTCTGAGCAGCA
Glyma.11G183700	TGA9	bZIP transcription factor	13.5	21.7	1.9	CTGAAAGGGGTGGCTGCTAA	ACGAATGCCCGAGTTCACAT

Supplementary Table S3. Normalized expression (heatmap) of soybean orthologs of Arabidopsis genes (Top BlastP hits) involved in cuticle formation. Fold-change (FC) expression values in 14A leaves, relative to WT, are provided for DE genes.

Annotation	Symbol	Arabidopsis Locus ID	Soybean Locus ID	WT	14A	FC
**LCFA Synthesis**						
Acyl carrier protein 1	ACP1	AT3G05020	*Glyma.07g042900*	12	13	
			Glyma.10g158500	669	904	1.5
Acyl carrier protein 4	ACP4	AT4G25050	*Glyma.05g201300*	4276	6378	1.6
			Glyma.08g008800	1928	2759	1.5
		AT1G54630	*Glyma.13g214600*	6993	9258	1.6
			Glyma.15g098500	1067	1408	1.5
Enoyl-ACP reductase	ENR1	AT2G05990	*Glyma.08g345900*	1128	1430	
			Glyma.11g101400	6638	6348	
			Glyma.12g027300	2125	2536	
			Glyma.18g156100	737	922	1.5
			Glyma.13g330100	783	860	
			Glyma.15g043900	29	18	
Fatty acyl-ACP thioesterase B	FATB	AT1G08510	*Glyma.04g151600*	1779	2665	1.5
			Glyma.04g197400		1	
			Glyma.04g197500	3	12	
			Glyma.05g012300	2862	2662	
			Glyma.06g168000			
			Glyma.06g168100	41	43	
			Glyma.06g211300	2503	2348	
			Glyma.10g268200	11	5	
			Glyma.17g120400	1898	1786	
			Glyma.20g122900	19	17	
Long-chain acyl-CoA synthetase 1	LACS1	AT5G57800	*Glyma.02g010300*	6,818	4,963	
			Glyma.03g221350	23,708	44,872	1.9
			Glyma.10g010800	14,777	17,588	
			Glyma.19g218300	24,777	47,772	1.9
**VLCFA Synthesis**						
ATP citrate lyase subunit B 2	ACLB-2	AT5G49460	*Glyma.07g239700*	870	1497	1.5
			Glyma.17g033600	169	152	
Very-long-chain 3-ketoacyl-CoA synthase 6	KCS6, CER6	AT1G68530	*Glyma.08g261100*	488	564	
			Glyma.10g241700	17	22	
			Glyma.10g274400	9167	17425	1.7
			Glyma.20g115500	9081	15899	1.6
Very-long-chain 3-ketoacyl-CoA synthase 11	KCS11	AT2G26640	*Glyma.02g001500*	2772	4257	
			Glyma.04g149300	103	84	
			Glyma.05g011100	1089	982	
			Glyma.06g214800	58	48	
			Glyma.10g001800	2474	5329	1.8
			Glyma.10g179400	5125	7612	
			Glyma.17g118700	3180	2610	
			Glyma.20g210900	337	633	1.7
Beta-ketoacyl reductase 1	KCR1	AT1G67730	*Glyma.11g245600*	2081	3098	1.6
			Glyma.11g245700	22	20	
			Glyma.18g011500	414	332	
			Glyma.18g011600	5650	8646	1.6
Very-long-chain 3-ketoacyl-CoA dehydratase	PAS2	AT5G10480	*Glyma.01g023400*			
			Glyma.08g279700	2129	4264	1.8
			Glyma.18g146900	1718	2903	1.6
Very-long-chain enoyl-CoA reductase isoform A	ECR, CER10	AT3G55360	*Glyma.02g273300*	2854	4830	1.5
			Glyma.14g043500	1586	1906	
**Wax Synthesis**						
ECERIFERUM 2; HXXXD-type acyl-transferase family protein	CER2	AT5G02890	*Glyma.11g037200*	371	590	1.6
Very-long-chain fatty acyl CoA reductase 3	CER4	AT4G33790	*Glyma.11g185000*	28	18	
			Glyma.11g185100	3,343	2,046	-1.6
			Glyma.11g185200	222	125	-1.8
			Glyma.11g185300	224	329	
			Glyma.12g087400	423	310	
			Glyma.12g183300	4	4	
			Glyma.12g183400	308	450	
			Glyma.13g317600	31	43	
			Glyma.13g317700	2	6	
Wax ester synthase/diacylglycerolacyltransferase (bifunctional)	WSD1	AT5G37300	*Glyma.06g291700*	33	16	
			Glyma.09g239600	1,045	1,090	
			Glyma.12g114400			
			Glyma.13g046600	1,214	985	
			Glyma.13g295900	33	25	
			Glyma.18g257900	4	6	1.8
			Glyma.18g258100			
			Glyma.19g046000	6,028	4,511	
Very-long-chain aldehyde decarbonylase	CER1	AT1G02205	*Glyma.03g101200*	3,071	1,261	-2.4
			Glyma.03g101700	313	243	
			Glyma.03g101800	18,705	31,121	1.7
			Glyma.03g102000			
			Glyma.07g114200	2,347	1,593	-1.5
			Glyma.07g121500	105	70	
Very-long-chain aldehyde decarbonylase	CER3	AT5G57800	*Glyma.13g091200*	9,714	14,002	
			Glyma.17g069100	21,456	35,416	1.7
**Cutin Synthesis**						
Glycerol-3-phosphate acyltransferase 2	GPAT2	AT1G02390	*Glyma.03g221100*	2	2	
			Glyma.14g167400	264	123	-2.2
			Glyma.19g218100	28	31	
Glycerol-3-phosphate acyltransferase 8	GPAT8	AT5G57800	*Glyma.03g008300*	574	900	1.6
			Glyma.07g069700	6,024	6,118	
Cutin synthase 2; GDSL-like Lipase/Acylhydrolase family protein	CUS2	AT5G33370	*Glyma.03g252600*	11	14	
			Glyma.03g252800	153	91	
			Glyma.05g116300	3,924	2,737	
			Glyma.09g241400	142	95	
			Glyma.10g168400	2	3	
			Glyma.10g168500			
			Glyma.13g044500	707	444	-1.6
			Glyma.13g045100	2,531	1,973	
			Glyma.18g254800	268	153	-1.8
			Glyma.19g050000	5,934	4,516	
			Glyma.19g050800	3,498	2,455	
			Glyma.19g050900	92	61	
			Glyma.19g051000	2,585	1,782	
			Glyma.19g051100	21,105	13,642	
			Glyma.19g250100	2	1	
			Glyma.19g250400	954	631	
			Glyma.20g221200	160	167	
**Transport of Cuticular Lipids**						
Acyl-CoA-binding protein 6	ACBP6	AT1G31812	*Glyma.04g122900*	3153	4631	1.7
			Glyma.09g214500	2338	3506	1.7
ABC transporter G family member 11	ABCG11	AT1G17840	*Glyma.08g070700*	88	72	
			Glyma.08g070800	1478	1666	
			Glyma.08g070900	441	774	
			Glyma.08g070967	7	4	
			Glyma.08g071033	4	0	
			Glyma.08g071100	228	211	
			Glyma.08g071200	2	2	
			Glyma.09g160000	5754	6607	
			Glyma.13g042900	36	41	
			Glyma.13g043000	633	959	
			Glyma.13g043500	234	172	-1.6
			Glyma.13g043600	0	0	
			Glyma.13g043800	228	147	-1.5
			Glyma.13g044000	164	157	
			Glyma.16g209400	3298	3931	
ABC transporter G family member 15	ABCG15/CER5	AT3G21090	*Glyma.03g135200*	71	23	
			Glyma.03g135300	0	0	
			Glyma.03g135375	0	0	
			Glyma.03g135525	366	273	-1.5
			Glyma.10g113802	209	201	
			Glyma.11g093800	1	5	
			Glyma.11g093900	3777	4570	
			Glyma.12g020300	4	2	
			Glyma.12g020400	4447	5224	
			Glyma.19g137700	2	0	
Lipid-transfer protein 1	LTP1	AT2G38540	*Glyma.01g130100*	0	0	
			Glyma.01g132600	2682	2112	
			Glyma.03g035600	3126	2112	
			Glyma.03g040100	4015	11611	2.3
			Glyma.03g040300	1246	1686	
			Glyma.03g040400	6	14	
			Glyma.09g278000	3925	1630	
			Glyma.18g210900	10129	8573	
			Glyma.18g214500	0	0	
Lipid-transfer protein 5	LTP5	AT3G18280	*Glyma.02g088900*	7109	7914	
			Glyma.05g002200	928	1218	
			Glyma.07g096700	1224	997	
			Glyma.09g181100	2250	1872	
			Glyma.10g121000	0	0	
			Glyma.10g132500	2271	5256	2.0
			Glyma.18g294200	1254	1848	
			Glyma.19g002300	741	768	
GPI-anchored lipid transfer protein 1	LTPG1	AT1G27950	*Glyma.05g147700*	1715	1035	
			Glyma.11g251100	4160	8122	1.9
			Glyma.18g005800	6391	15016	2.4
GPI-anchored lipid transfer protein 2	LTPG2	AT3G43720	*Glyma.13g075700*	6394	5367	
			Glyma.20g049800	2051	3869	1.7
**Regulation of Cuticle Development**						
Transcription factor MYB94	MYB94	AT3G47600	*Glyma.04g170100*	266	522	1.5
			Glyma.05g027000	418	888	1.6
			Glyma.06g193600	374	685	
			Glyma.17g099800	1,604	3,620	1.8

Supplementary Table S4. Expression of soybean orthologs of Arabidopsis genes (Top BlastP hits) that regulate stomatal development. Fold-change (FC) expression values in 14A leaves, relative to WT.

Gene (Glyma 2.0)	FC	Arabidopsis Ortholog	Symbol	Annotation
Glyma.03g167600	-1.5	AT1G04110	SDD1	STOMATAL DENSITY AND DISTRIBUTION 1 (SDD1); subtilisin-like protease SBT1.2
Glyma.05g051400				
Glyma.17g133400				
Glyma.17g133500				
Glyma.19g168700	-1.6			
Glyma.08g168400	-1.6	AT1G34245	EPF2	EPIDERMAL PATTERNING FACTOR 2
Glyma.15g258600	-1.6			
Glyma.13G365900	-1.7	AT1G80080	TMM	TOO MANY MOUTHS; leucine-rich repeat (LRR) family protein
Glyma.13g208300	-1.5	AT3G06120	MUTE	Transcription factor MUTE
Glyma.15g104600	-1.6			
Glyma.04g238400	-1.7	AT5G53210	SPCH	Transcription factor SPEECHLESS
Glyma.06g125500	-2.0			
Glyma.13g040100	-2.0			
Glyma.14g160700				
Glyma.09g152400		AT5G62230	ERL1	Leucine-rich repeat (LRR) receptor-like serine/threonine-protein kinase ERECTA-like 1
Glyma.10g242300				
Glyma.16g203300	-1.6			
Glyma.20g151800				
